# Nanophosphor-Based Contrast Agents for Spectral X-ray Imaging

**DOI:** 10.3390/nano9081092

**Published:** 2019-07-30

**Authors:** Kevin Smith, Matthew Getzin, Josephine J. Garfield, Sanika Suvarnapathaki, Gulden Camci-Unal, Ge Wang, Manos Gkikas

**Affiliations:** 1 Department of Chemistry, University of Massachusetts Lowell, Lowell, MA 01854, USA; 2 Department of Biomedical Engineering, Rensselaer Polytechnic Institute, Troy, NY 12180, USA; 3 Biomedical Engineering and Biotechnology Program, University of Massachusetts Lowell, Lowell, MA 01854, USA; 4 Department of Chemical Engineering, University of Massachusetts Lowell, Lowell, MA 01854, USA

**Keywords:** nanophosphors, CT imaging, X-ray contrast agents, spectral CT, lanthanide nanoparticles, computed tomography, rare earth metals

## Abstract

Lanthanide-based nanophosphors (NPhs) are herein developed as contrast agents for spectral X-ray imaging, highlighting the chemical, macromolecular and structural differences derived from ligand exchange on computed tomography (CT) and solvent dispersibility. Taking advantage of the ability of spectral X-ray imaging with photon-counting detectors to perform image acquisition, analysis, and processing at different energy windows (bins), enhanced signal of our K-edge materials was derived, improving sensitivity of CT imaging, and differentiation between water, tumor-mimic phantoms, and contrast materials. Our results indicate that the most effective of our oleic acid-stabilized K-edge nanoparticles can achieve 2–4x higher contrast than the examined iodinated molecules, making them suitable for deep tissue imaging of tissues or tumors. On the other hand, ligand exchange yielding poly(acrylic acid)-stabilized K-edge nanoparticles allows for high dispersibility and homogeneity in water, but with a lower contrast due to the high density of the polymer grafted, unless further engineering is probed. This is the first well-defined study that manages to correlate NPh grafting density with CT numbers and water dispersibility, laying the groundwork for the development of the next generation CT-guided diagnostic and/or theranostic materials.

## 1. Introduction

Cancer detection is a great challenge in medicine [1,2,3,4,5,6,7,8]. For many cancer types being undetectable from early symptoms or blood tests, and often detected at late stages (such as ovarian, pancreatic, and non-small lung cancer), medical imaging emerges as the most promising tool for broad spectrum cancer screening and detection. X-ray computed tomography (CT) is one of the most widely used imaging procedures in diagnostic medicine due to its many advantages including deep tissue penetration, fast scanning times, patient comfort, and high resolution [9,10,11]. Small water-soluble iodinated molecules, such as diatrizoate and iohexol (Omnipaque), can effectively absorb X-rays and are routinely used in vivo for contrast-enhanced CT studies. However, they suffer from short circulation lifetime (~10 min) and pose potential renal toxicity [12,13,14], while a large amount of contrast agent is required for CT imaging (~30 g of iodine in humans for oral administration; intravenous administration is also used). Another notable disadvantage is the relatively low density (4.93 g/mL) and low K-edge of iodine (33 keV). On the other hand, CT contrast agents comprising metal elements of high atomic number (high-Z) such as tantalum, gold, platinum, and iridium [15,16], as well as rare earth metals such as lutetium, europium, ytterbium, erbium, gadolinium, etc., have been proven as a powerful tool to address this challenge [17,18,19]. The very low abundance and higher cost of the first category, however, restricts their possible future utilization in hospital settings, considering the high quantity of contrast agent needed for CT imaging.

Rare earth metals [20,21], on the other hand, are abundant in the earth’s crust, have a low cost, a density range between 5.24–9.94 g/mL, and a K-edge of 48–63 keV, both much higher than iodine. Synthetically prepared rare-earth fluoride nanoparticles called “nanophosphors” (La-NPhs) offer the advantage to be dually utilized as CT contrast agents, as well as photon upconversion molecules (UCNPs) with directed NIR emission (when excited with a 980 nm laser), make them suitable for bioimaging [17,18,19,22,23,24,25,26,27,28,29,30]. These contrast agents have exhibited excellent imaging capabilities, acceptable safety profiles, and long circulation in vivo [19,29]. The NIR emission provided by the La-NPhs overcomes the problems of insufficient tissue light transmission, scattering, and tissue autofluorescence, provides high signal-to-background ratio [30], and allows for effective photoluminescence imaging [17,18,19,20,21,22,23,24,25,26,27,28,29,30]. However, there are critical gaps in current lanthanide contrast agents (and future theranostic materials), related to detection limitations of only cancer cells, detection close to the skin surface, and animals studies restricted only to mice (shallow tissue penetration) [17,18,19,20,21,22,23,24,25,26,27,28,29,30]. Tuning synthetic parameters of NPhs and instrumentation techniques to allow for the detection of deep tissue contrast in larger animals (such as rats, rabbits, etc.), so as to bring contrast agents closer to hospital instrumentation settings is therefore a great challenge. To achieve that, a thorough study on the X-ray contrast ability of various NPhs, as well as the effect of NPh ligand exchange (and the outcome of different grafting densities on the NPh surface) on CT contrast and water dispersibility is mandatory.

Spectral micro-CT using photon-counting detectors (PCDs) has been proven as an excellent tool to increase separability of contrast agents from soft tissues and biological fluids [31,32,33]. Photon-counting CT is a rapidly emerging form of CT that uses a standard polychromatic X-ray source and PCDs [34,35,36]. It provides energy-dependent information on individual X-ray photons, leading to accurate material decomposition and simultaneous quantification of multiple contrast generating materials. Maximizing contrast in water, biological fluids, and tumor-mimic phantoms, as well as achieving significant separability in those, would allow for NPh dosage prediction per specific volume of phantom (e.g., μg–mg of contrast agent per cm^3^ of phantom), which can later be expanded to NPh dosage prediction in cancerous tissue. That would be highly informative for the design and development of future diagnostic materials, since it could assist surgeons to remove tumors more effectively, and could possibly permit for detection of secondary cancers, defined as metastasized tumors that are usually return 4–10 year after the treatment of cancer/surgical resection.

Towards that direction, NPh parameters such as the % of grafted ligand (stabilizer), its effect on contrast ability, as well as its impact on hydrophilicity and cell viability, should be taken into consideration for the advancement of CT imaging. Finding the golden ratio between those is thus crucial and a huge focus in our studies. Herein, we leverage advanced photon-counting X-ray CT techniques [32,33,35,36,37,38,39,40,41,42,43,44,45] to achieve deep tissue imaging, empowered by lanthanide-based contrast agents (K-edge materials) that can provide with enhanced contrast in aqueous solutions, as well as in tumor-mimic phantoms. Our synthetic NPhs comprise either a high metal-to-stabilizer ratio for development of oily NPh formulations with higher contrast than iodinated molecules, or a high (water-soluble) polymer grafting density onto the nanocrystal surface that allows for high dispersibility and homogeneity is aqueous solutions. Together with long in vivo circulation time, these nanomaterials could be utilized as a high-performance CT contrast agents for local delivery and detection of cancer, or other diseases, as well as for bimodal imaging (e.g., CT/PET).

## 2. Materials and Methods

### 2.1. Materials

YbCl_3_, GdCl_3_, EuCl_3_, LuCl_3_, ErCl_3_, and TaCl_5_, (purity > 99.99%) were obtained from Alfa Aesar (Haverhill, MA, USA). Octadecene, oleic acid, ammonium fluoride, and formamide were purchased from TCI Chemicals (Portland, OR, USA). Poly(acrylic acid) 1800 g/mol and sodium diatrizoate were purchased from Sigma-Aldrich (St. Louis, MO, USA).

### 2.2. Synthesis of Oleic Acid Stabilized Nanophosphors (OA-Stabilized NPhs)

Oleic acid (15 mL, 47.5 mmol) and octadecene (23 mL) were added in the glove box in a 100 mL dry flask containing LuCl_3_ (422 mg, 1.5 mmol, 281.33 mg/mmol). The flask was capped and transferred to the N_2_ line. The solution was heated to 160 °C under N_2_ to yield a homogeneous clear solution, which was then cooled to room temperature. Subsequently, a methanolic solution (15 mL) containing NH_4_F (0.22 g, 6 mmol) and NaOH (0.15 g, 3.75 mmol) was added slowly dropwise, and the solution was gradually heated to 220 °C under N_2_ to remove residual water and low boiling point impurities. The temperature was then increased to 300 °C and remained at that temperature for 3 h. The yellow colloidal solution was then cooled to room temperature, and the precipitated NPhs were collected via centrifugation (10,000 rpm, 20 minutes, 20 °C). OA-stabilized NPhs were finally washed twice with warm ethanol (55 °C) to remove oleic acid, sodium oleate, and unreacted salts, and finally dried under vacuum. High yields were obtained for Ta-, Lu-, and Yb-NPhs (300–400 mg); moderate yields for Eu- and Er-NPhs (100–270 mg); the lower yield was obtained for Gd-NPhs (60 mg). For the doped materials, different molar ratios of lanthanide chlorides were used (provided in x/y % molar ratio) to a total content of 1.5 mmol.

### 2.3. Water-Dispersible Poly(acrylic acid) Stabilized Nanophosphors (PAA-Stabilized NPhs)

The ligand/stabilizer exchange was performed following a slightly different literature method [29]. PAA (0.5 g, 1800 g/mol) was dissolved in formamide (8 mL) and added to a 50 mL flask. The solution was equilibrated to 110 °C with vigorous stirring. A 2 mL toluene dispersion (previously sonicated for 20 min and vortexed) of OA-stabilized NPhs (30 mg) was then injected, and the mixture stirred until complete removal of toluene. The mixture was then gradually heated to 210 °C under N_2_ for 2 h, until obtaining a clear brown solution (yellow drops of material condense to the adapter). After cooling the solution, excess deionized water was added leading to a precipitation. The PAA-stabilized NPhs were collected by centrifugation (10,000 rpm, 20 min, 20 °C), and were further washed twice with DI water to remove unbound PAA. For low PAA grafting materials (enhanced CT contrast agents), the same recipe was used but 0.1 g of PAA (1800 g/mol) was added.

### 2.4. Cell Studies

The different PAA-stabilized NPhs were tested with a cancer cell line for their cytocompatibility over one day exposure. A human lung epithelial carcinoma cell line (A549) was used in the cell culture study. The cells were grown in a 96-well plate and exposed to the NPhs at 1000 μg/ mL, 100 μg/mL, 10 μg/mL and 0 μg/mL (control test) concentrations for 24 h. The PAA stabilizer was also tested at the same concentrations. Three replicates per NPh concentration were performed. The metabolic activity of the cells upon interaction with the NPhs at different concentrations was evaluated using the alamarBlue^®^ assay following the manufacturer’s protocol. The fluorescence intensities for the alamarBlue^®^ test were measured using a SpectraMax plate reader at 560 nm/590 nm (Ex/Em) wavelengths. The metabolic activity was recorded as a percentage (%) referenced to the 0 μg/mL NPh concentration (control). The statistical analyses were performed with GraphPad Prism (La Jolla, CA, USA). One-way ANOVA tests were executed for comparison of different concentrations of NPhs. Statistical significances were given as follows: * *p* < 0.05, ** *p* < 0.01 and *** *p* < 0.001.

### 2.5. Gelatin Phantoms

Gelatin derived from porcine skin (Sigma-Aldrich, gel strength 300, type A) was dissolved in water at 7 wt% at 50 °C and 0.5 mL of that solution was added to a 3 mL vial and left to cool down initially at room temperature and then in the fridge for 20 min (this initial layer allows for contrast agent deposition in the center of the vial). The gel was then removed from the refrigerator, the vial was foiled on top, and a disposable plastic microspatula (with both ends cut with a blade) was placed in the center of the vial (after puncturing a small area in the center of the foil) in order to be used as a mold for the contrast agent addition. 1.5 mL of the slightly warm gelatin solution was sequentially injected through a small side hole in the foil, and the gelatin was placed in the refrigerator for 20 min to solidify. The microspatula was then removed carefully, leaving a cylindrical mold for material deposition, and a NPh-dispersion (mixed with a small amount of gelatin so as to avoid diffusion) was added in the mold. The material was placed in the refrigerator for 20 min to solidify. Sequentially, another 0.5 mL of the slightly warm gelatin solution was added on top of the solidified sample, left at room temperature to cool down, and then placed into the refrigerator for 20 min. The tumor-mimic phantom contained overall embedded NPhs (in μg/mL) in 2.5 mL of solid gelatin.

### 2.6. Characterization

Fourier-transform infrared spectroscopy (FTIR) spectra were recorder using a Thermo Scientific Nicolet 4700 FTIR (Waltham, MA, USA) with a Smart Orbit attenuated total reflectance (ATR) accessory in the frequency range 4000–400 cm^−1^ with a resolution of 4 cm^−1^ with 32 scans. Thermogravimetric analysis (TGA) was recorded on a TA Instrument Discovery thermal analyzer (New Castle, DE, USA) at a heating rate of 20 °C/min under N_2_ atmosphere from 25–600 °C. For TGA analysis, samples were vacuum-dried at 70 °C for 12 h prior analysis. Transmission electron microscopy (TEM) images were collected at room temperature using a FEI Tecnai Spirit 12 (Hillsboro, OR, USA), with an acceleration voltage of 200 kV (UMass Medical School Core Facilities). For sample preparation, OA- and PAA-stabilized NPh dispersions were prepared at 0.6 mg/mL in ethanol and water respectively, filtered through a Millipore 0.45 mm filter, and a drop of material was deposited on a Formvar/Carbon 200 mesh copper grid (Ted Pella, 01800-F). For aqueous sample preparation, 10 μL of sample were deposited on the grid, and the excess solution was removed with filter paper (after 2 min). The hydrodynamic size and distribution of our NPhs was measured by dynamic light scattering (DLS) at 25 °C on a Malvern Zetasizer Nano ZS90 (Southborough, MA, USA) with a He/Ne laser (633 nm) at 173° collecting optics. PAA-stabilized NPh dispersions were prepared at 0.06 mg/mL in water (filtered) and filtered through a Millipore 0.45 μm filter (Burlington, MA, USA) prior to measurements. Polystyrene spheres of 100 nm were used as a standard. A z- and number-average effective hydrodynamic diameters of 104.6 ± 1.4 nm and 100.4 ± 1.6 nm were found respectively. Ethanol dispersions of OA-NPhs at 0.06 mg/mL filtered with a 0.45 μm filter, showed low counts without appearance of a form factor. Similar results were obtained with a 0.8 μm filter, denoting higher aggregation in that solvent. Data were analyzed by Malvern Dispersion Technology Software 4.20 (Southborough, MA, USA).

### 2.7. Imaging Studies

The Medipix All-Resolution Scanner (MARS, manufactured and distributed by MARS Bioimaging Ltd., Christchurch, New Zealand) located on the main campus of Rensselaer Polytechnic Institute in Troy, NY was employed for imaging experiments with the test contrast materials. Two imaging protocols were used, reflecting the outer diameter of the sample holder (also known as phantom) used in this investigation. The solvent-solute mixtures were placed in custom-machined acrylic tubes (6.4 mm outer diameter, 4.0 mm inner diameter). These tubes were then placed in a larger acrylic holder (~51 mm outer diameter) with 6 spaces for the acrylic tubes. The scanning parameters for this phantom were as follows: 120 kVp tube voltage, 22 µA tube current, 283.1 mm source-to-detector distance (SDD), 223.1 mm source-to-object distance (SOD), 250 msec integration time, 58 mm field-of-view (FOV), 1.96 mm aluminum (Al) beam filtration, and energy thresholds of 28.6, 41.0, 53.6, and 65.1 keV. The tumor-mimicking phantoms were solidified in ~15 mm diameter glass vials. The scanning parameters for the tumor-mimicking phantom were as follows: 120 kVp tube voltage, 19 µA tube current, 266.1 mm SDD, 223.1 mm SOD, 250 msec integration time, 32 mm FOV, 1.96 mm Al beam filtration, and same energy thresholds as previous. All of the attenuation properties reported were obtained using the MARS Vision software pre-installed on the scanner.

## 3. Results and Discussion

### 3.1. Synthesis and Characterization of OA-Stabilized NPhs

**Scheme 1:**
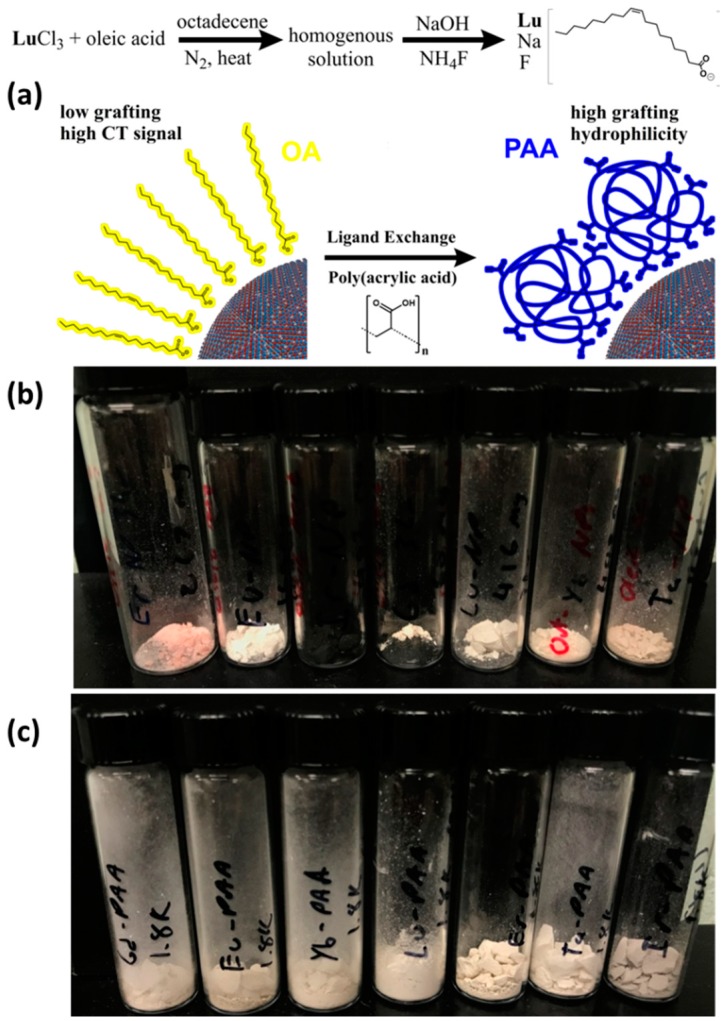
(**a**) Schematic representation of OA-stabilized nanophosphors (NPhs) and ligand-exchange to water-dispersible poly(acrylic acid) (PAA)-stabilized NPhs; (**b**) From left to right: OA-stabilized Er-NPhs, Eu-NPhs, Ir-NPhs, Gd-NPhs, Lu-NPhs, Yb-NPhs, and Ta-NPhs; (**c**) PAA-stabilized NPhs with high PAA grafting density.

OA-stabilized La-NPhs were prepared by thermal treatment of lanthanide chlorides (LaCl_3_), NaOH, NH_4_F, and oleic acid in octadecene (Scheme 1). Different OA-stabilized NPhs were prepared, leading to 11 different K-edge materials, providing significant information between various contrast materials. In the study, we included Ta as well for comparison, due to its high K-edge and density, though it is not a lanthanide. The NhPs were consisted either from one metal ion or a combination of two (lanthanide dopants at x/y % mole ratio); Yb (100), Gd (100), Eu (100), Er (100), Lu (100), Ta (100), as well as Yb/Eu (98/2), Gd/Eu (50/50), Yb/Er (98/2), Yb/Lu (50/50), and Gd/Lu (50/50) NPhs were synthesized, as shown in Table 1. The incorporation of OA into the nanocrystal structure was revealed by FTIR. OA-coated Lu-NPhs show two bands at 1560 cm^−1^ (v_as_: COO^−^) and 1464 cm^−1^ (v_s_: COO^−^) attributed to the oleate ion bound onto the nanocrystal surface [27,46,47] (red line in Figure S1), unlike free OA, where the characteristic carboxylic peak is shown at 1710 cm^−1^ (black line). In addition, bands at 2924 and 2852 cm^−1^, shown in both samples are assigned to the asymmetric (v_as_) and symmetric (v_s_) stretching vibrations of the methylene (–CH_2_) groups in the long alkyl chain of OA. The synthesized OA-stabilized NPhs could be dispersed in chloroform, THF, and dichloromethane, while the dispersibility was moderate at 30% ethanol, a compatible solvent system for our acrylic-made sample holders utilized for micro-CT imaging (Figure S2).

### 3.2. CT Imaging of OA-Stabilized NPh Contrast Agents

**Figure 1:**
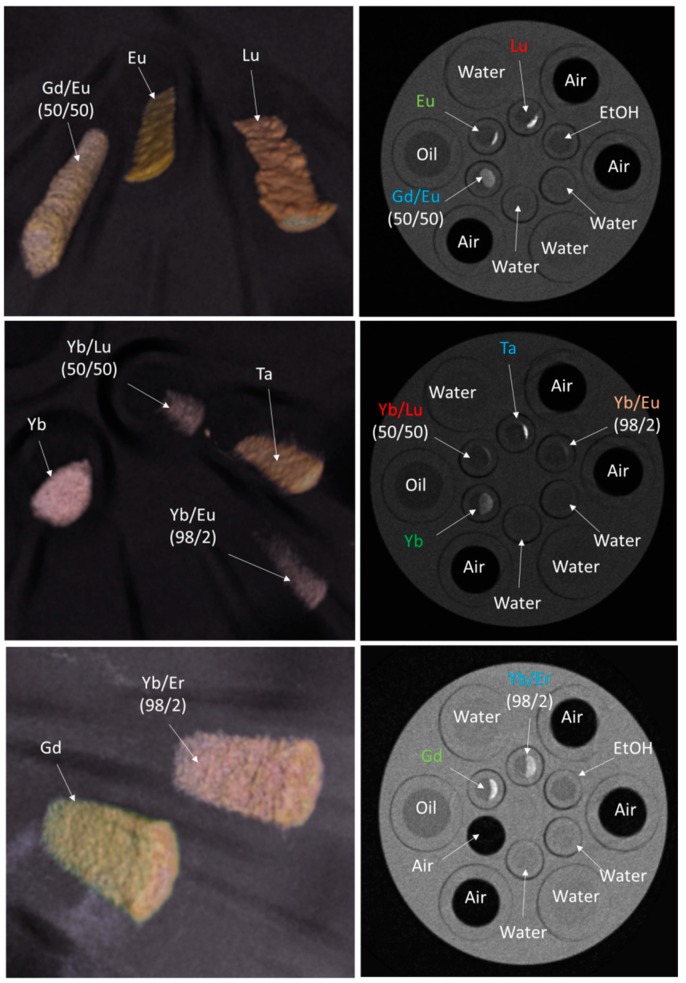
Micro-CT images of different OA-stabilized nanophosphors (NPhs) at 30 mg/mL in 30% ethanol, including either 100% metals (Eu, Lu, Yb, Gd, Ta) or combination of metal dopants [Gd/Eu (50:50), Gd/Lu (50:50), Yb/Eu (98:2), Yb/Er (98:2), and Yb/Lu (50:50)]. Unlike the reported fluorescence upconversion trend of doped La-NPhs, pure metals showed higher CT contrast.

High contrast was achieved by using Yb, Gd, Eu, Lu, and Ta (100%), as well as Gd/Eu (50:50), Gd/Lu (50:50), Yb/Eu (98:2), and Yb/Er (98:2) in 30% ethanol at 30 mg/mL (Figure 1), while Yb/Lu (50/50) had a lower contrast. Lu and Ta had the highest contrast, something which can be explained by the high density (9.8 and 16.7 g/mL respectively) and high K-edge values (63.3 and 67.3 keV respectively) of those metals. By selecting identically sized regions of interests (ROI) within all the materials (Figure S2), and by subtracting the value of the solvent from the specific energy bin (spectral CT allows you to locate specifically your inorganic material according to its K-edge), solvent-corrected attenuation coefficient values of 1.020 ± 0.140, 0.657 ± 0.087, 0.450 ± 0.107, 0.268 ± 0.024 and 0.206 ± 0.015 cm^−1^ were obtained for OA-stabilized Ta, Lu, Eu, Gd, and Yb-NPhs respectively at 30 mg/mL in 30% ethanol (Table 1 and Table S1). Er-NPhs had a low dispersibility in that solvent system and thus could not be measured. For the dopant metals, only OA-stabilized Gd/Eu (50/50) and Gd/Lu (50/50) had high values (0.227 ± 0.025, and 0.225 ± 0.021 cm^−1^ respectively), very close to diatrizoate (0.234 ± 0.019 cm^−1^). These CT values obtained were similar (Yb,Gd) or higher (x2-4 times higher in the case of Ta, Lu, Eu) than those obtained for the iodinated contrast agent, sodium diatrizoate (0.234 ± 0.019 cm^−1^) at 30 mg/mL in water (18 mg of I/mL, ~60% of the molecular mass of diatrizoate is attributed to iodine), a contrast agent which is often administered to patients for CT imaging (Figure S3). To perform a direct comparison, however, a ~4% grafting of OA in the nanocrystal core should be considered for OA-stabilized NPhs (Figure S4), yielding 30 mg/mL × 0.96 = 28.8 mg of metal/mL compared to 18 mg of I/mL obtained for sodium diatrizoate.

To obtain absolute values however, the attenuation coefficient values were divided by the metal content (in mg/mL). Results showed 0.016 ± 0.004 cm^-1^ for [Eu], 0.023 ± 0.003 cm^−1^ for [Lu], and 0.035 ± 0.005 cm^−1^ for [Ta], all comparable/higher than diatrizoate (0.013 ± 0.001 cm^−1^ for [I]). It is important to note that iodinated molecules are fully soluble in the water, while NPhs are colloidal formulations. Even in that case though, a high CT signal is obtained for some of our NPhs, showing contrast potency similar/higher to iodinated molecules. Additional information on the homogeneity and dispersibility of the nanophosphors at the allowed (from sample holder’s material perspective: acrylic, Figure S2) solvent system was provided. High homogeneity and dispersibility was achieved by utilizing Yb (100%) and Gd (100%), as well as Yb/Er (98:2) and Gd/Eu (50:50) in 30% ethanol (Figure 1). The majority of NPhs formed a strip of material in the sample holder during scanning, due to the low dispersibility of OA-stabilized NPhs in that solvent system. OA-stabilized Gd-NPhs and Yb-NPhs seemed to be the most promising option for that solvent system, combining relatively high dispersibility/homogeneity with high contrast (though lower than Eu, Lu, and Ta), while Yb/Er (98:2) and Gd/Eu (50:50) from the dopant mixtures were also promising candidates (Figure 1).

Our results overall indicate that the most effective of our K-edge NPhs (Ta, Lu, Eu) can achieve high contrast, similar to that of iodinated molecules, making them suitable for deep tissue imaging. While iodine-based contrast agents are administered orally or intravenously (e.g., angiography) to provide contrast and circulate in the bloodstream for a short duration of time until being excreted (~10 min), our lanthanide-based contrast agents have been designed to cover longer circulation times, as those are needed for time-dependent detection of tumors or other diseases. Oily formulations of our contrast agents (in oleic, caprylic, or linolenic acid) could be developed and administered transdermally or locally to cancer-specific models of rodents for deep tissue imaging.

### 3.3. Ligand Exchange and Characterization of PAA-Stabilized NPhs

**Figure 2:**
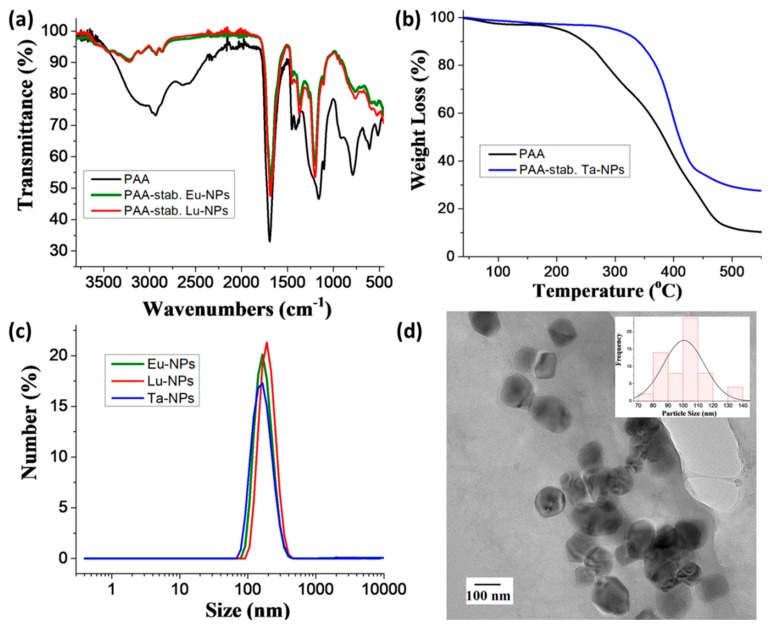
(**a**) FTIR, (**b**) TGA, and (**c**) dynamic light scattering (DLS) spectra of PAA-stabilized Lu-NPhs (red line), Eu-NPhs (green line), and Ta-NPhs (blue line) along with PAA (black line). The number-average diameter was 183.3 ± 27.7 nm, 184.5 ± 26.2 nm, and 166.6 ± 5.0 nm for PAA-stabilized Lu-, Eu-, and Ta-NPhs respectively; (**d**) TEM image of PAA-stabilized Eu-NPhs with a diameter of 101.9 ± 15.6 nm.

In order to increase hydrophilicity and modulate the synthesized NPhs for biologically-relevant environments, the stabilizer (ligand) was exchanged with poly(acrylic acid) (PAA), a widely used water-soluble polymer with pendant carboxylic groups in each monomeric unit (Scheme 1). PAA is listed as a food additive by FDA, and has been used for drug delivery, or as a stabilizer for a variety of other nanomaterials for in vivo imaging [29,30,48,49,50]. Different PAA-stabilized NPhs were obtained as shown in Table 2. The as-prepared PAA-stabilized NPhs were well-dispersed in water, even at high concentrations, and remained stable and homogenous for days, allowing for suitable use in biomedical and future clinical applications. The incorporation of PAA into the nanocrystal structure was revealed by FTIR. The characteristic C=O stretching peak for carboxylic acid, shown at 1695 cm^−1^ in the case of PAA (black line), was slightly shifted to 1688 cm^−1^ for all the PAA-stabilized NPhs (blue line, Lu-NPhs; red line, Eu-NPhs), while other known PAA peaks at 1455 cm^−1^ and 1410 cm^−1^ (symmetric stretching mode of –COO^−^ and bending mode of the PAA backbone) were also observed (Figure 2a and Figures S5–S10). It is important to highlight that unlike OA-coated particles, PAA has multiple carboxylic groups, that can either be found complexed onto the nanocrystal surface or as remain free as –COOH or –COO^−^ groups, depending on the pH. This allows for further complexation with specifically targeted drugs or other biomarkers for post-modification.

The amount of PAA attached covalently on the surface of the NPh cores was found to be 68–74% for all the examined NPhs (Table 2), as assessed by TGA (Figure 2b and Figures S11–S16). Small differences in the grafting density, as will be shown later, affect both the CT contrast and the solvent dispersibility. The polymer grafting density obtained in our study (~70%) was much higher than the values reported in literature for aqueous dispersion-yielding stabilizers, which range from 4–20% (azelaic acid ~7.5% [27]; PEG ~ 20% [19]; PAA ~ 4% [48]), with limited information to be provided for solvent dispersibility. The higher attachment/grafting of a water-soluble polymer onto the nanocrystal core should highly impact the water-dispersibility of the NPhs. As a result, La-NPh dispersions were dispersed even at high concentrations (>90 mg/mL, paste-like form). DLS measurements of our synthesized nanophosphors showed an effective hydrodynamic (z-average) diameter of 226.2 ± 15.1 nm, 216.0 ± 10.3 nm, and 184.6 ± 6.8 nm for PAA-stabilized Lu-, Eu-, and Ta-NPhs respectively (Figure 2c). TEM results of the PAA-stabilized Eu-NPhs showed spherical particles with a diameter of 101.9 ± 15.6 nm (Figure 2d), while Lu-NPhs and especially Ta-NPhs showed a tendency to form dimers or trimers after drying (Figure S17). Therefore, DLS was preferred over TEM for characterization of PAA-stabilized NPhs, since it depicts the actual swelling behavior of NPhs in aqueous solution. The obtained size values for PAA-stabilized NPhs seem to be in agreement with those obtained for OA-stabilized NPhs (before the ligand exchange), where spherical particles of 90.1 ± 14.7 nm, 77.0 ± 21.0 nm, and 65.6 ± 13.9 nm diameter were found for Eu-, Lu-, and Ta-NPhs respectively (Figure S18). PAA, unlike OA, is a random coil polymer with multiple ligands for complexation per macromolecule chain, and it was grafted at a higher extent (~70%). As a result, an increase in NPh diameter is expected.

### 3.4. CT Imaging of PAA-Stabilized NPh Contrast Agents

**Figure 3:**
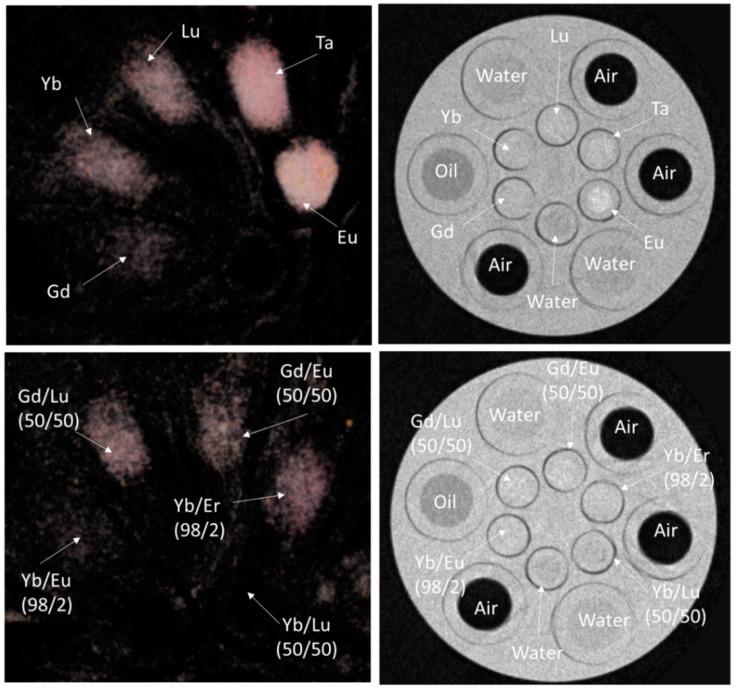
Micro-CT images of different PAA-stabilized NPhs at 90 mg/mL in water. The contrast shown in the images is derived from bin 1 and is only indicative of the water dispersibility of the samples (not the attenuation). The attenuation coefficient of those samples was measured from bins 4 or 5. Higher color strength represents attenuation coefficient intensity.

Higher contrast was achieved by using PAA-stabilized Ta, Lu, Er, and Eu-NPhs in water at 90 mg/mL (Figure 3), while moderate contrasting ability was obtained for Yb-NPhs and Gd-NPhs. From the dopant-materials, Yb/Er (98:2), Gd/Eu (50:50), and Gd/Lu (50:50) had high attenuation values, while lower contrast was observed for Yb/Eu (98:2) and Yb/Lu (50:50). Ta, Lu, Er, and Eu showed, in addition, the highest water dispersibility among all the materials, while the dopant-formulations had moderate dispersibility at the reported concentration. The ligand modification allowed for high NPhs dispersion in water (Figure 3), albeit a decrease in the contrast ability (defined as the provided contrast of the inorganic NPh core compared with the grafting density of the stabilizer) should be expected, in comparison with OA-stabilized NPhs, due to the highly bound polymeric ligand onto the nanocrystal surface (Scheme 1). Unlike OA, where only one –COOH is available for attachment (OA has an elongated hydrophobic tail), PAA has a much higher density of carboxylic groups available for complexation (every monomeric unit has a pendant –COOH), as well as a higher molecular weight (1800 g/mol for PAA vs. 254 g/mol for OA). Both these reasons allow PAA, as a random coil, to wrap around the nanocrystal core and coordinate with the metal to a high extent, achieving high grafting density (~70%) for all the formulations.

As a result, the material gains in water dispersibility, which is crucial for biomedical and clinical applications, but pays a penalty in contrast due to the high polymer grafting density (Table 2 and Table S2). The best results were obtained for PAA-stabilized Lu (0.036 ± 0.014 cm^−1^), Er (0.067 ± 0.019 cm^−1^), and Eu-NPs (0.086 ± 0.021 cm^−1^), while from the dopants, Gd/Eu (50/50) and Gd/Lu (50/50) had relatively high values (0.043 ± 0.021 cm^−1^ and 0.041 ± 0.021 cm^−1^ respectively). However, the highest value obtained (for PAA-stabilized Eu-NPhs) was ~1/3 of that of diatrizoate. Interestingly, PAA-stabilized Eu-NPhs had the highest contrast and simultaneously the highest dispersibility in water, granting this material as a potential clinical imaging probe. Since, finding the golden ratio between high contrast in X-ray imaging and high water dispersibility is crucial and a key thrust area in our lab, further material design and engineering was probed to enhance the CT signal.

NPhs with a lower grafting density were sequentially designed, compared with the previous highly dense materials (~70% PAA), in order to increase the metal content, and thus the contrast ability, taking into account the importance of water dispersibility. PAA-stabilized Lu-NPhs’ with a PAA grafting density of ~18% (Figure S19) showed a high attenuation coefficient in the range of 0.303 ± 0.017 cm^−1^ (Figure S20 and Table 2, last row). The obtained value managed to exceed diatrizoate at 30 mg/mL in water (0.234 ± 0.019 cm^−1^), granting our synthesized NPh material with a lower grafting density as a very promising contrast agent. Considering an 18% polymer grafting density, 73.8 mg of metal/mL were obtained for PAA-stabilized Lu-NPhs’ compared to 18 mg of I/mL for sodium diatrizoate. The absolute attenuation coefficient value for our low grafted material was 0.004 ± 0.000 cm^−1^ for [Lu’], compared with 0.013 ± 0.001 cm^−1^ for [I] (diatrizoate). The FTIR spectrum of 18% grafted PAA-stabilized Lu-NPhs’ showed the characteristic C=O stretching peak for carboxylic acid to be slightly shifted to 1688 cm^−1^ for the case of new PAA-stabilized Lu-NPs (Figure S20), while the bound polymer (asterisk) onto the nanocrystal surface was shown at 1565 cm^−1^ (v_as_: COO^−^) and 1445 cm^−1^ (v_s_: COO^–^). Similar peaks have been also reported in the literature [48,49,50].

The NPhs had additionally high homogeneity and moderate-to-good dispersibility (Figure S20), highlighting once again the two opposing trends (CT contrast vs aqueous dispersibility). Efforts in our lab are currently orientated towards achieving even higher dispersibility, but with a similar enhanced contrast. It is important to note that iodinated molecules are fully soluble in the media, while the PAA-stabilized NPhs are aqueous dispersants. Understanding the fundamental differences derived from ligand exchange, with effects in both the contrast agent dispersibility and CT numbers, is of paramount importance. Bringing in symphony those opposite trends, prior to animal studies, is thus crucial for nanomaterial and contrast agents engineering. This is the first well-defined study that manages to correlate grafting density of the NPhs (by TGA) with CT values and solvent dispersibility. The high contrast provided for our low grafting density materials could be utilized for intravenous, oral, or transdermal (local) administration and delivery of contrast agent to tumors (or other specific targeted tissues), either by itself, or combined with anti-cancer drugs (such as Doxorobucin and Taxol [51] or immunotherapeutics [3,52,53]), associating the enhanced contrast with therapeutic efficacy (theranostic material).

### 3.5. Cell Viability of PAA-Stabilized NPhs

**Figure 4:**
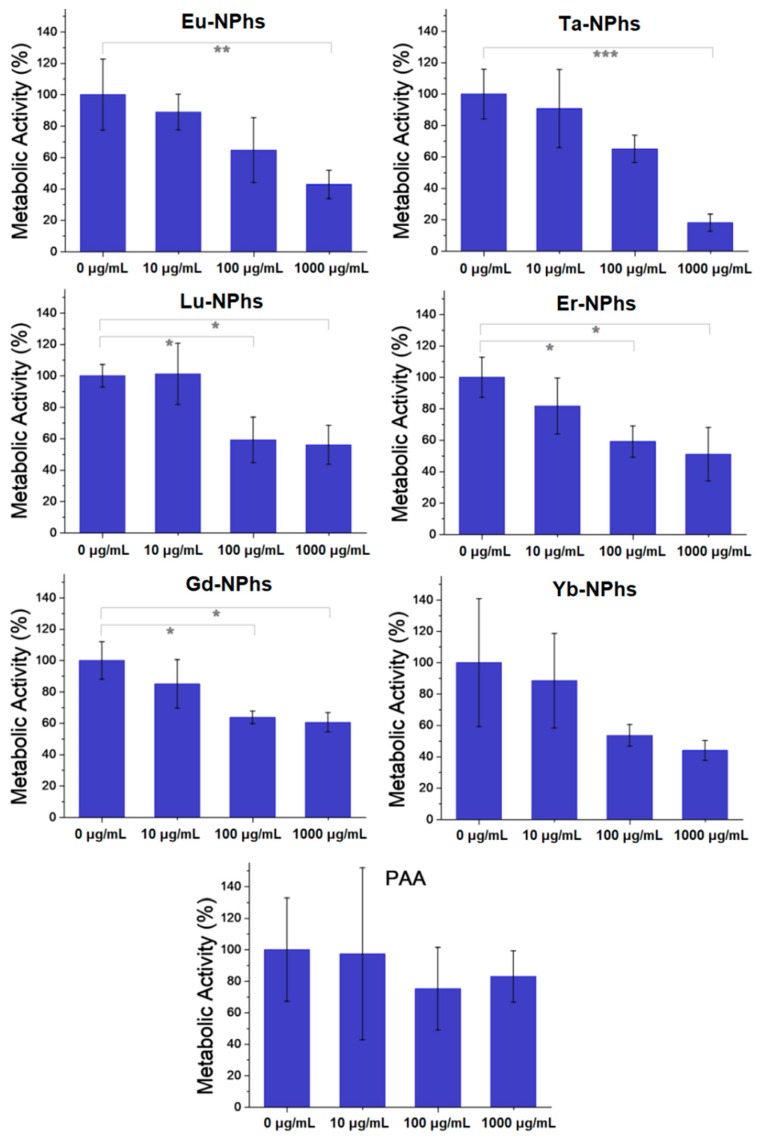
Metabolic activity (%) of the human lung epithelial carcinoma cell line A549 with PAA-stabilized NPhs. Cells were incubated with 0, 10, 100, and 1000 μg/mL of PAA-stabilized NPhs (Eu, Ta, Lu, Er, Gd, Yb) as well as with the stabilizer (PAA) itself for 24 h at 37 °C; *p < 0.05, **p < 0.01 and ***p < 0.001.

To determine the viability of our PAA-stabilized contrast agents, cell studies were performed using a human lung epithelial carcinoma cell line (A549). The fluorescent signal generated from the assay (the non-toxic, cell-permeable compound resazurin from the alamarBlue^®^ reagent is converted to the fluorescent reporter resorufin in metabolically active cells) measures proliferation in living cells [54,55]. A low metabolic activity (low % signal) could imply damage on cancer cells. No significant difference in the metabolic activity of the cells was observed for the PAA stabilizer (~97%) at 10 μg/mL, while ~75–83% of the metabolic activity was noticed at 100 and 1000 μg/mL respectively (Figure 4). The PAA-stabilized lanthanide NPhs showed cytocompatibility profiles in agreement with published work at similar concentrations with other cancer-related cell lines (KB, HeLa) [18,29]. Some of the NPhs showed safer cytocompatibility profiles, with a statistically significant absence in the metabolic activity up to 100 μg/mL, prompting those as safe to be used at that concentration window. That is the concentration range at which we are planning to utilize our NPhs for local deep tissue imaging (CT imaging results in phantoms at those dosages are shown below).

Figure 4 demonstrates the effect of PAA-stabilized Eu-NPhs on the metabolic activity of the A549 cells after 24 h of exposure. The increasing amount of Eu-NPhs did not significantly decrease the metabolic activity and cytocompatibility at 10 μg/mL and 100 μg/mL concentrations. At the highest concentration of 1000 μg/mL however, the metabolic activity was decreased, as expected. A549 cells behaved similarly against exposure to the same concentrations of PAA-stabilized Ta-NPhs. Regarding the PAA-stabilized Lu-NPhs, the metabolic activity of A549s was maintained constant when they were exposed to 10 μg/mL. On the contrary, when the amount of NPhs was further increased to 100 μg/mL and 1000 μg/mL, the negative effects of Lu-NPhs on the metabolic activity of the cells was observed. There was no change in the metabolic activity when the A549s were exposed to PAA-stabilized Er-NPhs at 10 μg/mL. However, the data showed a significant decrease in the metabolic activity upon exposure at higher concentrations (100 μg/mL and 1000 μg/mL). The same trend was similarly observed for PAA-stabilized Gd-NPhs. There was a significant decrease in the metabolic activity of the cells upon exposure to 100 μg/mL and 1000 μg/mL of PAA-stabilized Gd-NPhs compared to the no particles condition (0 μg/mL). For the PAA-stabilized Yb-NPhs, there was no significant difference in the metabolic activity of the A549s when they interacted with any of the concentrations of the Yb-NPhs (0–1000 μg/mL). Although there was a decreasing trend for the cellular metabolic activity, PAA-stabilized Yb-NPhs did not significantly damage the cells even at high concentrations, denoting that the Yb-materials are the most compatible with that particular cell line. The PAA polymer itself did not damage significantly the cells at any of the concentrations (0–1000 μg/mL). The results overall demonstrated that the PAA-stabilized NPhs, prepared by ligand exchange of OA-stabilized NPhs, have good water dispersibility and acceptable cytocompatibility (for Eu, Ta, and Yb) with A549 cells up to 100 μg/mL (and below 1000 μg/mL), suggesting that they could be used for deep tissue imaging of cancerous tissues or tracking of other diseases at that concentration regime.

### 3.6. Contrast Efficacy of NPhs Embedded into Phantoms

**Figure 5:**
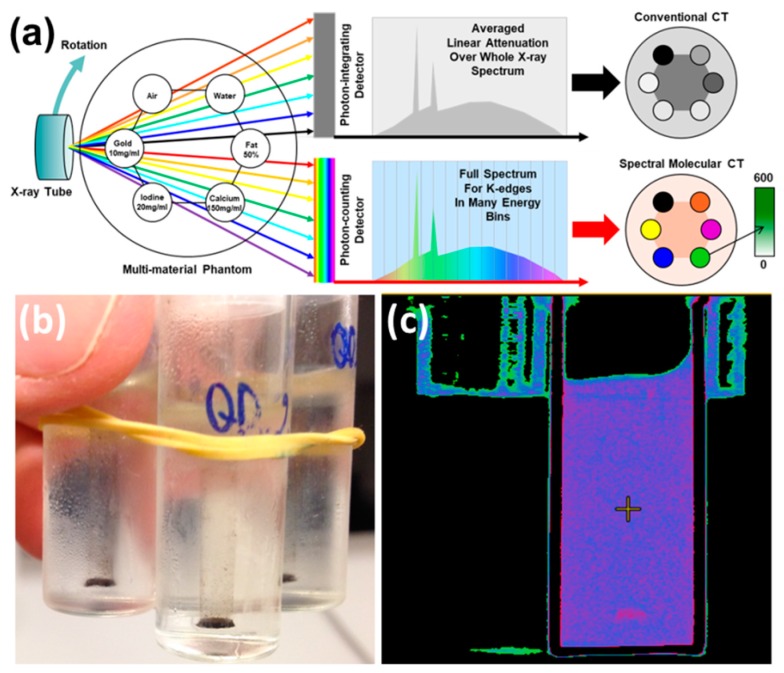
(**a**) Schematic of a spectral CT with photon-counting detectors (PCDs) compared with a conventional CT. (**b**) A dispersion of 160 μg PAA-stabilized Lu-NPhs’ (front image; 18% grafting density) embedded onto a 1 × 1 × 2.5 cm^3^ gelatin, tumor-mimic phantom, imitating desired material accumulation into tumors. (**c**) CT scan of the front image phantom from b in our spectral CT scanner showing high material separability. Black: vial with nothing loaded (air); Pink: hydrogel media; Intense pink: contrast agent.

The contrast ability of our NPhs was finally studied in tumor-mimic, hydrogel phantoms in order to explore the contrast ability. Since different materials have differing X-ray attenuation profiles as a function of X-ray photon energy, characterizing the energy distribution of the transmitted beam with PCDs allows for the detection of different contrast agents. In the same way that color imaging expands the dimension of measured visible photons from 1D (grayscale) to 3D (RGB), spectral CT provides the means for expanding the dimension of measured X-ray photons (Figure 5a). The enabling technology of the MARS spectral micro-CT scanner is the X-ray photon-counting camera, the Medipix3RX, which is an assembly of multiple cadmium-zinc-telluride (CZT) detector modules, and offers differentiation between biological fluids, tissues, and contrast agents. This camera is capable of sorting the incoming photons according to their energy levels by energy thresholds applied to detector pixels and processed by state-of-the-art charge-summing circuitry. Given that X-ray photons interact with matter in a material- and energy-dependent manner, PCDs, and the MARS scanner specifically, are highly suitable for preclinical and clinical spectral X-ray imaging.

Results showed that our synthesized nanophosphors can give excellent contrast at low concentrations when embedded in gelatin phantoms, and imaged by our spectral CT scanner, imitating desired material accumulation in tumors (Figure 5b,c). High contrast was obtained by using 160 μg of our 18% grafted PAA-stabilized Lu-NPs’ dispersion in 2.5 mL of solid gelatin (1 × 1 × 2.5 cm^3^), which was utilized as a tumor-mimic phantom. This demonstrates the feasibility to image and spectrally distinguish our NPhs using spectral X-ray imaging. This is of paramount importance, since it could allow for estimations of the amount of contrast agent required per area of interest (cm^3^), such as the size of the tumor. In addition, approximations can be made for the detection of smaller tumor sizes, when our material is attached, allowing for potential detection of early cancers, tracking of cancer metastases (secondary cancers), and quantification of therapeutic responses [3,51,52,53,56].

## 4. Conclusions

Lanthanide-based nanophosphors are herein developed as contrast agents for spectral X-ray imaging, highlighting the differences derived from ligand exchange on solvent dispersibility and CT contrast. Taking advantage of the ability of spectral X-ray imaging with photon-counting detectors to perform image acquisition at different energy levels, enhanced signal and high material separation of our K-edge materials was derived, improving sensitivity for CT imaging, and promoting differentiation between water and tumor-mimic phantoms. The results indicate that the most effective of our utilized OA-stabilized K-edge nanophosphors can achieve 2–4x higher contrast than the utilized iodinated molecules, making them suitable for local delivery and imaging of tumors in oily formulations, while ligand exchange yielding PAA-stabilized K-edge nanophosphors allowed for high dispersibility and homogeneity in water, but with a lower contrast due to the higher density of the polycarboxylic material grafted. Synthesizing, however, nanophosphor probes with $\frac{1}{4}$ of the previously high grafted PAA (18%), finally allows for CT contrast higher than the examined iodinated agent, with good water dispersibility. Designing materials that could combine enhanced CT contrast with high water dispersibility, and cell viability, could enable the development of molecular diagnostic probes for CT imaging and promote high-end novel cancer theranostics by coupling those with cancer chemo/immuno therapeutic drugs.

## Figures and Tables

**Table 1:** Computed tomography (CT) values and characteristics of the different synthesized OA-stabilized nanophosphors (NPhs).

Yb%	Gd%	Er%	Eu%	Lu%	Ta%	I%	Atten. Coeff. ^1^ (cm^−1^)	Hmg ^2^	D (g/mL)	K-edge (keV)
100	-	-	-	-	-	-	0.206 ± 0.015	+	6.9	61.3
-	100	-	-	-	-	-	0.268 ± 0.024	+	7.9	50.3
-	-	100	-	-	-	-	-	-	6.9	57.5
-	-	-	100	-	-	-	0.450 ± 0.107	-	5.2	48.5
-	-	-	-	100	-	-	0.657 ± 0.087	-	9.8	63.3
-	-	-	-	-	100	-	1.020 ± 0.140	-	16.7	67.4
-	-	-	-	-	-	100 ^3^	0.234 ± 0.019	+	4.9	33.2
98	-	2	-	-	-	-	0.181 ± 0.014	+	-	-
98	-	-	2	-	-	-	0.173 ± 0.023	-	-	-
	50	-	-	50	-	-	0.225 ± 0.021	-	-	-
50	-	-	-	50	-	-	0.141 ± 0.031	-	-	-
-	50	-	50	-	-	-	0.227 ± 0.025	+	-	-

^1^ Attenuation coefficient values subtracted from the solvent (30% ethanol for OA-stabilized NPhs and 100% water for diatrizoate). ^2^ Hmg: homogeneity in the solvent system. ^3^ Diatrizoate at 30 mg/mL in water. Unlike the NPhs which showed a K-edge (sudden increase in attenuation) between bin 4 (Eu, Gd: 53.6–65.1 keV) and bin 5 (Yb, Lu, Ta: 65.1–120.0 keV), the K-edge information of iodinated molecules was obtained from bin 2 (28.6–41.0 keV). OA-stabilized Er-NPhs had very low dispersibility in 30% ethanol and thus were not measured.

**Table 2:** CT values and characteristics of the different PAA-stabilized NPhs.

Yb%	Gd%	Er%	Eu%	Lu%	Ta%	I%	Atten. Coeff. ^1^ (cm^−1^)	Hmg ^2^	K-edge (keV)	PAA (%) wt Loss ^3^
100	-	-	-	-	-	-	0.027 ± 0.020	+	61.3	68.1 ± 0.4
-	100	-	-	-	-	-	0.028 ± 0.021	+	50.3	74.3 ± 1.0
-	-	100	-	-	-	-	0.067 ± 0.019	+	57.5	71.8 ± 1.3
-	-	-	100	-	-	-	0.086 ± 0.021	+	48.5	72.0 ± 1.3
-	-	-	-	100	-	-	0.036 ± 0.014	+	63.3	71.3 ± 0.2
-	-	-	-	-	100	-	0.029 ± 0.013	+	67.4	68.9 ± 0.3
-	-	-	-	-	-	100 ^4^	0.234 ± 0.019	+	33.2	-
98	-	2	-	-	-	-	0.033 ± 0.015	+	-	-
98	-	-	2	-	-	-	0.005 ± 0.013	+	-	-
	50	-	-	50	-	-	0.041 ± 0.021	+	-	-
50	-	-	-	50	-	-	0.009 ± 0.013	+	-	-
-	50	-	50	-	-	-	0.043 ± 0.021	+	-	-
-	-	-	-	100 ^5^	-	-	0.303 ± 0.017	+	63.3	18.1 ± 0.6

^1^ Attenuation coefficient values subtracted from the solvent (100% water for all). ^2^ Hmg: homogeneity at 90 mg/mL water. Unlike the NPhs which showed a K-edge between bin 4 (Eu, Gd: 53.6–65.1 keV) and bin 5 (Yb, Lu, Ta: 65.1–120.0 keV), the K-edge information of iodinated molecules was obtained from bin 2 (28.6–41.0 keV). ^3^ Obtained by TGA. For the unbound PAA polymer, the % weight loss was 87.2 ± 0.5 with a decomposition temperature of 350 °C. ^4^ Diatrizoate at 30 mg/mL in water. ^5^ Synthesized Lu-NPhs’ with a ~18% grafting density.

## Supplementary Materials

The following are available online at https://www.mdpi.com/2079-4991/9/8/1092/s1, Figure S1: FTIR spectra of OA and OA-stabilized Lu-NPhs, Figure S2: acrylic sample holder photo and definition of region of interest (ROI) in CT, Figure S3: micro-CT images of iodinated molecules, Figure S4: typical TGA spectrum of OA-stabilized NPhs, Figures S5–S10: FTIR spectra of PAA-stabilized Gd-, Yb-, Ta-, Eu-, Er-, and Lu-NPhs, Figures S11–S16: TGA spectra of PAA-stabilized Gd-, Yb-, Ta-, Eu-, Er-, and Lu-NPhs, Figures S17–S18: TEM images of OA- and PAA-stabilized Eu-, Lu- and Ta-NhPs, Figures S19–S20: TGA spectrum, FTIR, and micro-CT image of low grafted PAA’-stabilized Lu-NPhs, Tables S1–S2: Attenuation coefficient values of all the synthesized OA- and PAA-stabilized NPhs as well as those of the carrier solvent.

## References

[1] Markou A., Liang Y., Lianidou E. (2011). Prognostic, therapeutic, and diagnostic potential of microRNAs in lung cancer. Clin. Chem. Lab. Med..

[2] Mastoraki S., Strati A., Tzanikou E., Chimonidou M., Politaki E., Voutsina A., Psyrri A., Georgoulias V., Lianidou E. (2018). ESR1 methylation: A liquid biopsy–based epigenetic assay for the follow-up of patients with metastatic breast cancer receiving endocrine treatment. Clin. Cancer Res..

[3] Schmid D., Park C.G., Hartl C.A., Subedi N., Cartwright A.N., Puerto R.B., Zheng Y., Maiarana J., Freeman G.J., Wucherpfennig K.W. (2017). T cell-targeting nanoparticles focus delivery of immunotherapy to improve antitumor immunity. Nat. Commun..

[4] Joseph C.S., Patel R., Neel V.A., Giles R.H., Yaroslavsky A.N. (2014). Imaging of ex vivo nonmelanoma skin cancers in the optical and terahertz spectral regions optical and Terahertz skin cancers imaging. J. Biophotonics.

[5] Melikechi N., Markushin Y., Connolly D., Lasue J., Ewusi-Annan E., Makrogiannis S. (2016). Age specific classification of discrimination of blood plasma samples of healthy and ovarian cancer prone mice using laser-induced breakdown spectroscopy. Spectrochim. Acta Part B At. Spectrosc..

[6] Gaudiuso R., EboEwusi-Annan E., Melikechi N., Sun X., Liu B., Campesato L.F., Merghoub T. (2018). Using LIBS to diagnose melanoma in biomedical fluids deposited on solid substrates: Limits of direct spectral analysis and capability of machine learning. Spectrochim. Acta Part B At. Spectrosc..

[7] Spirou S.V., Costa Lima S.A., Bouziotis P., Vranješ-Djurić S., Efthimiadou E. Κ., Laurenzana A., Barbosa A.I., Garcia-Alonso I., Jones C., Jankovic D. (2018). Recommendations for in vitro and in vivo testing of magnetic nanoparticle hyperthermia combined with radiation therapy. Nanomaterials.

[8] Toniolo G., Efthimiadou E.K., Kordas G., Chatgilialoglu C. (2018). Development of multi-layered and multi-sensitive polymeric nanocontainers for cancer therapy: In vitro evaluation. Sci. Rep..

[9] Kalender W.A. (2006). X-ray computed tomography. Phys. Med. Biol..

[10] Momose A., Takeda T., Itai Y., Hirano K. (1996). Phase-contrast X-ray computed tomography for observing biological soft tissues. Nat. Med..

[11] Paulus M.J., Gleason S.S., Kennel S.J., Hunsicker P.R., Johnson D.K. (2000). High resolution X-ray computed tomography: An emerging tool for small animal cancer research. Neoplasia.

[12] Haller C., Hizoh I. (2002). Radiocontrast-induced renal tubular cell apoptosis: Hypertonic versus oxidative stress. Investig. Radiol..

[13] Haller C., Hizoh I. (2004). The cytotoxicity of iodinated radiocontrast agents on renal cells in vitro. Investig. Radiol..

[14] de Vries A., Custers E., Lub J., van den Bosch S., Nicolay K., Grüll H. (2010). Block-copolymer-stabilized iodinated emulsions for use as CT contrast agents. Biomaterials.

[15] Popovtzer R., Agrawal A., Kotov N.A., Popovtzer A., Balter J., Carey T.E., Kopelman R. (2008). Targeted gold nanoparticles enable molecular CT imaging of cancer. Nano Lett..

[16] Janib S.M., Moses A.S., MacKay J.A. (2010). Imaging and drug delivery using theranostic nanoparticles. Adv. Drug Deliv. Rev..

[17] He M., Huang P., Zhang C., Hu H., Bao C., Gao G., He R., Cui D. (2011). Dual phase-controlled synthesis of uniform lanthanide-doped NaGdF_4_ upconversion nanocrystals via an OA/ionic liquid two-phase system for in vivo dual-modality imaging. Adv. Funct. Mater..

[18] Zhou J., Zhu X., Chen M., Sun Y., Li F. (2012). Water-stable NaLuF_4_-based upconversion nanophosphors with long-term validity for multimodal lymphatic imaging. Biomaterials.

[19] Liu Y., Ai K., Liu J., Yuan Q., He Y., Lu L. (2012). A high-performance ytterbium-based nanoparticulate contrast agent for in vivo X-ray computed tomography imaging. Angew. Chem. Int. Ed..

[20] Davies A., Lewis D.J., Watson S.P., Thomas S.G., Pikramenou Z. (2012). pH-controlled delivery of luminescent europium coated nanoparticles into platelets. Proc. Natl. Acad. Sci. USA.

[21] Lewis D.G., Pikramenou Z. (2014). Lanthanide-coated gold nanoparticles for biomedical applications. Coord. Chem. Rev..

[22] Heer S., Kömpe K., Güdel H.U., Haase M. (2004). Highly efficient multicolour upconversion emission in transparent colloids of lanthanide-doped NaYF_4_ nanocrystals. Adv. Mater..

[23] Kramer K.W., Biner D., Frei G., Güdel H.U., Hehlen M.P., Luthi S.R. (2004). Hexagonal sodium yttrium fluoride based green and blue emitting upconversion phosphors. Chem. Mater..

[24] Zhang Y.-W., Sun X., Si R., You L.-P., Yan C.-H. (2005). Single-crystalline and monodisperse LaF_3_ triangular nanoplates from a single-source precursor. J. Am. Chem. Soc..

[25] Boyer J.C., Vetrone F., Cuccia L.A., Capobianco J.A. (2006). Synthesis of colloidal upconverting NaYF_4_ nanocrystals doped with Er^3+^, Yb^3+^ and Tm^3+^, Yb^3+^ via thermal decomposition of lanthanide trifluoroacetate precursors. J. Am. Chem. Soc..

[26] Nyk M., Kumar R., Ohulchanskyy T.Y., Bergey E.J., Prasad P.N. (2008). High contrast in vitro and in vivo photoluminescence bioimaging using near infrared to near infrared up-conversion in Tm^3+^ and Yb^3+^ doped fluoride nanophosphors. Nano Lett..

[27] Chen Z., Chen H., Hu H., Yu M., Li F., Zhang Q., Zhou Z., Yi T., Huang C.N. (2008). Versatile synthesis strategy for carboxylic acid-functionalized upconverting nanophosphors as biological labels. J. Am. Chem. Soc..

[28] Xiong L., Chen Z., Tian Q., Cao T., Xu C., Li F. (2009). High contrast upconversion luminescence targeted imaging in vivo using peptide-labeled nanophosphors. Anal. Chem..

[29] Xiong L., Yang T., Yang Y., Xu C., Li F. (2010). Long-term in vivo biodistribution imaging and toxicity of polyacrylic acid-coated upconversion nanophosphors. Biomaterials.

[30] Chen G., Shen J., Ohulchanskyy T.Y., Patel N.J., Kutikov A., Li Z., Song J., Pandey R.K., Agren H., Prasad P.N. (2012). (a-NaYbF_4_:Tm^3+^)/CaF_2_ core/shell nanoparticles with efficient near-infrared to near-infrared upconversion for high-contrast deep tissue bioimaging. ACS Nano.

[31] Roessl E., Proksa R. (2007). K-edge imaging in X-ray computed tomography using multi-bin photon counting detectors. Phys. Med. Biol..

[32] He P., Wei B., Cong W., Wang G. (2012). Optimization of K-edge imaging with spectral CT. Med. Phys..

[33] He P., Yu H., Bennett J., Ronaldson P., Zainon R., Butler A., Butler P., Wei B., Wang G. (2013). Energy-discriminative performance of a spectral micro-CT system. J. X-ray Sci. Technol..

[34] Taguchi K., Iwanczyk J.S. (2013). Vision 20/20: Single photon counting X-ray detectors in medical imaging. Med. Phys..

[35] Cammin J., Kappler S., Weidinger T., Taguchi K. Photon-counting CT: Modeling and compensating of spectral distortion effects. Proceedings of the SPIE.

[36] Cammin J., Kappler S., Weidinger T., Taguchi K. (2016). Evaluation of models of spectral distortions in photon-counting detectors for computed tomography. J. Med. Imaging.

[37] Wang G., Lin T.H., Cheng P., Shinozaki D.M. (1993). A general cone-beam reconstruction algorithm. IEEE Trans. Med. Imaging..

[38] Xu Q., Yu H., Mou X., Zhang L., Hsieh J., Wang G. (2012). Low-dose X-ray CT reconstruction via Dictionary Learning. IEEE Trans. Med. Imaging..

[39] Feng P., Cong W.X., Wei B.A., Wang G. (2014). Analytic comparison between X-Ray fluorescence CT and K-edge CT. IEEE Trans. Biomed. Eng..

[40] Meng B., Cong W.X., Xi Y., De Man B., Wang G. (2016). Energy window optimization for X-ray K-edge tomographic imaging. IEEE Trans. Biomed. Eng..

[41] Zhang Y., Mou X., Wang G., Yu H. (2017). Tensor-based dictionary learning for spectral CT reconstruction. IEEE Trans. Med. Imaging.

[42] Meng B., Cong W., Xi Y., De Man B., Yang J., Wang G. (2017). Model and reconstruction of a K-edge contrast agent distribution with an X-ray photon-counting detector. Opt. Express.

[43] Getzin M., Garfield J.J., Rundle D.S., Krueger U., Gkikas M., Wang G. (2018). Increased separability of K-edge nanoparticles by photon-counting detectors for spectral micro-CT. J. X-ray Sci. Technol..

[44] Cormode D.P., Si-Mohamed S., Bar-Ness D., Sigovan M., Naha P.C., Balegamire J., Lavenne F., Coulon P., Roessl E., Bartels M. (2017). Multicolor spectral photon-counting computed tomography: In vivo dual contrast imaging with a high count rate scanner. Sci. Rep..

[45] Si-Mohamed S., Cormode D.P., Bar-Ness D., Sigovan M., Naha P.C., Langlois J.B., Chalabreysse L., Coulon P., Blevis I., Roessl E. (2017). Evaluation of spectral photon counting computed tomography K-edge imaging for determination of gold nanoparticle biodistribution in vivo. Nanoscale.

[46] Nara M., Tanokura M. (2008). Infrared spectroscopic study of the metal-coordination structures of calcium-binding proteins. Biochem. Biophys. Res. Commun..

[47] Cotton F.A., Wilkinson G., Murillo C.A., Bochmann M. (1988). Advanced Inorganic Chemistry.

[48] Liu B., Chen Y., Li C., He F., Hou Z., Huang S., Zhu H., Chen X., Lin J. (2015). Poly(acrylic acid) modification of Nd^3+^-sensitized upconversion nanophosphors for highly efficient UCL imaging and pH-responsive drug delivery. Adv. Funct. Mater..

[49] Wang L., Zhang Y., Zhu Y. (2010). One-pot synthesis and strong near-infrared upconversion luminescence of poly(acrylic acid)-functionalized YF_3_:Yb^3+^/Er^3+^ nanocrystals. Nano. Res..

[50] Celebi S., Erdamar A.K., Sennaroglu A., Kurt A., Yagci H.A. (2007). Synthesis and characterization of poly(acrylic acid) stabilized cadmium sulfide quantum dots. J. Phys. Chem. B.

[51] Iatrou H., Dimas K., Gkikas M., Tsimblouli C., Sofianopoulou S. (2014). Polymersomes from polypeptide containing triblock Co- and terpolymers for drug delivery against pancreatic cancer: Asymmetry of the external hydrophilic blocks. Macromol. Biosci..

[52] Stephan M.T., Moon J.J., Um S.H., Bershteyn A., Irvine D.J. (2010). Therapeutic cell engineering with surface-conjugated synthetic nanoparticles. Nat. Med..

[53] Moon J.J., Huang B., Irvine D.J. (2012). Engineering nano- and microparticles to tune immunity. Adv. Mater..

[54] Wei Z., Chen L., Thompson D.M., Montoya L.D. (2012). Effect of particle size on in vitro cytotoxicity of titania and alumina nanoparticles. J. Exp. Nanosci..

[55] Watanabe M., Yoneda M., Morohashi A., Hori Y., Okamoto D., Sato A., Kurioka D., Nittami T., Hirokawa Y., Shiraishi T. (2013). Effects of Fe_3_O_4_ magnetic nanoparticles on A549 cells. Int. J. Mol. Sci..

[56] Gkikas M., Peponis T., Mesar T., Hong C., Avery R.K., Roussakis E., Yoo H.-J., Parakh A., Patino M., Sahani D.V. (2019). Systemically administered hemostatic nanoparticles for identification and treatment of internal bleeding. ACS Biomater. Sci. Eng..

