# Supplementary material for: Nanophosphor-Based Contrast Agents for Spectral X-ray Imaging
Source: Nanomaterials (Basel). 2019 Jul 30;9(8):1092. doi: 10.3390/nano9081092 (PMC6723483; doi:10.3390/nano9081092)
Supplement: Supplementary file 1 [file nanomaterials-09-01092-s001.pdf]

Article

Nanophosphor-Based Contrast Agents for Spectral X-ray Imaging

Kevin Smith [1], Matthew Getzin [2], Josephine J. Garfield [1], Sanika Suvarnapathaki [3], Gulden Camci-Unal [4], Ge Wang [2,*] and Manos Gkikas [1,*]

[1] Department of Chemistry, University of Massachusetts Lowell, Lowell, MA 01854, USA
[2] Department of Biomedical Engineering, Rensselaer Polytechnic Institute, Troy, NY 12180, USA
[3] Biomedical Engineering and Biotechnology Program, University of Massachusetts Lowell, Lowell, MA 01854, USA
[4] Department of Chemical Engineering, University of Massachusetts Lowell, Lowell, MA 01854, USA
* Correspondence: wangg6@rpi.edu (G.W.), manos_gkikas@uml.edu (M.G.); Tel.: +1-518-276-4259 (G.W.), +1-978-934-3245 (M.G.)

Received: 5 July 2019; Accepted: 27 July 2019; Published: date

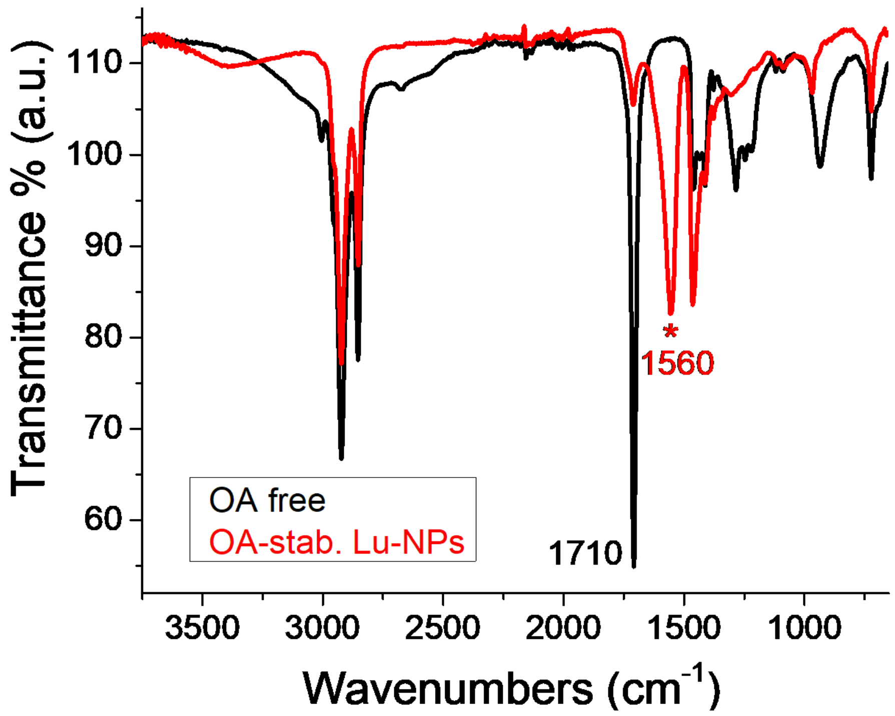

Figure S1. FTIR spectra of OA (black line) and OA-stabilized Lu-NPhs (red line). Results showed two bands at 1560 cm^{-1} (v_{as}: COO^{-}) and 1464 cm^{-1} (v_s: COO^{-}) attributed to the oleate ion bound on the nanocrystal surface, unlike free oleic acid where the characteristic carboxylic peak at 1710 cm^{-1} is shown.

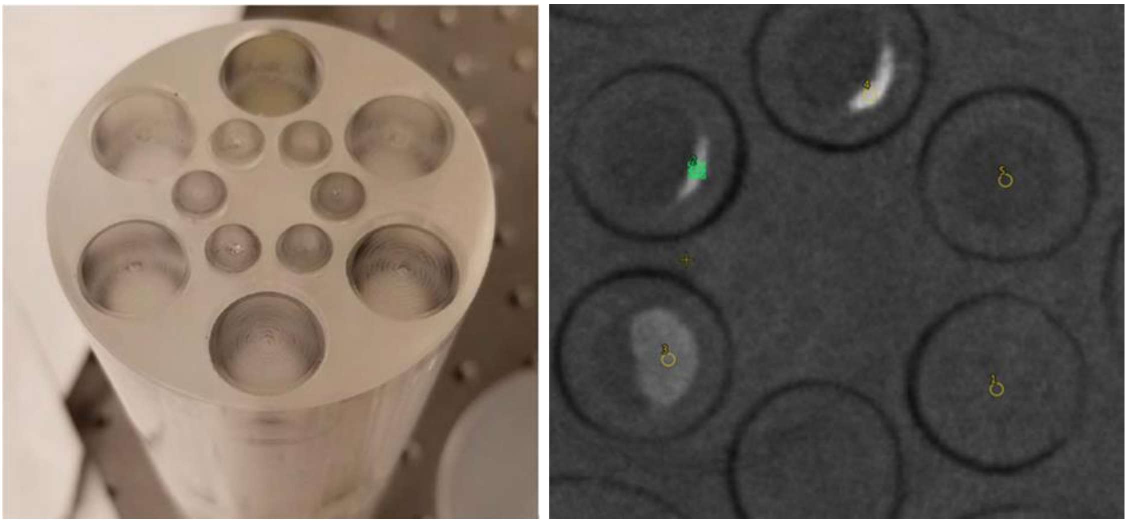

Figure S2. (Left) The 4.8 cm diameter acrylic phantom utilized can hold up to 12 liquid mixtures. The larger diameter tubes hold ~2 mL while the smaller tubes hold ~500 µL. (Right) Micro-CT images showing the collected regions of interest (ROI) of identical size for all the materials were selected and subtracted from the solvent yielding solvent-corrected attenuation coefficient values for the different K-edge materials. From the green mark clockwise: OA-stabilized Eu-NPhs, OA-stabilized Lu-NPhs, ethanol, water, and OA-stabilized Gd/Eu (50/50)-NPhs.

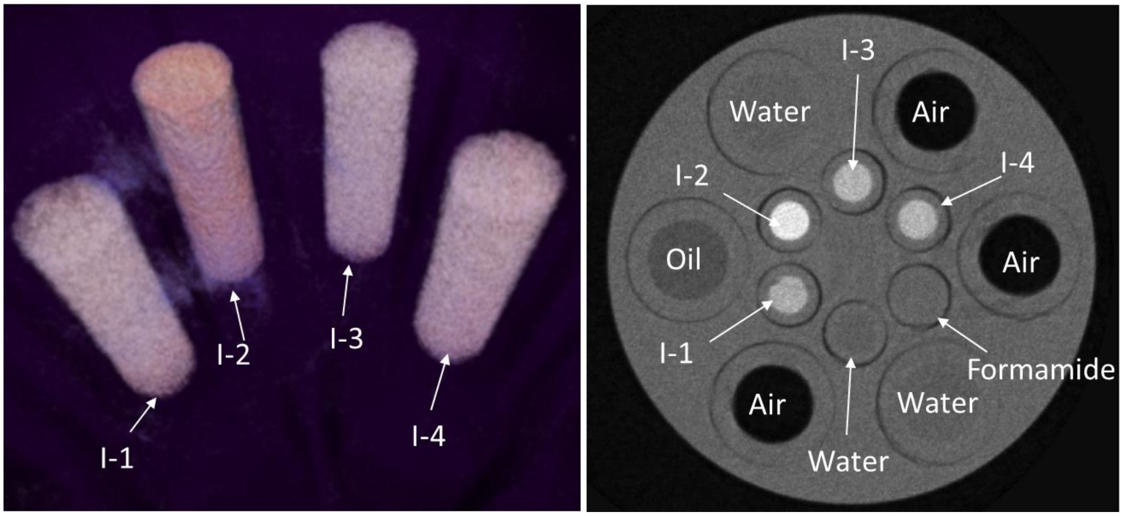

Figure S3. Micro-CT images and obtained contrast from iodinated molecules. I-1: Sodium diatrizoate at 30 mg/mL in water (18 mg of I/mL); I-2: Iohexol (Omnipaque) at 30 mg of I/mL in water; I-3: Chemically modified diatrizoic acid at 30 mg/mL in water; I-4: Diatrizoic acid at 30 mg/mL in formamide.

NPhs	Weight Loss (%)
**OA-stab. Eu**	3.81 ± 0.11
**OA-stab. Ta**	4.35 ± 0.05
**OA-stab. Lu**	11.36 ± 0.14

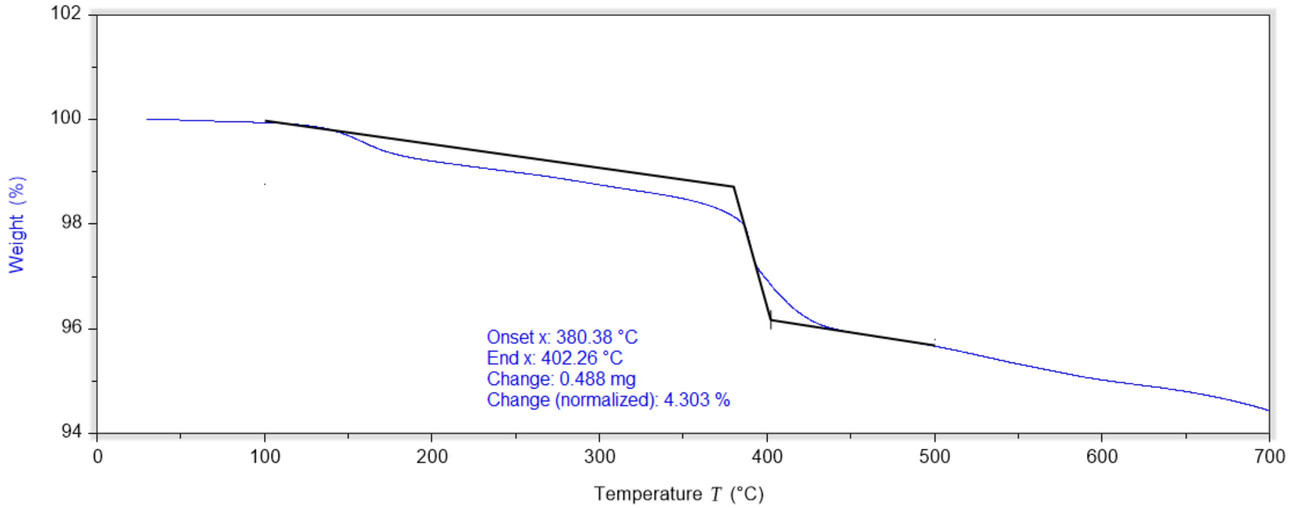

Figure S4. Typical TGA spectrum of OA-stabilized **Ta-NPhs** showing a ~4 wt% grafting.

Table S1. Attenuation coefficient values of different synthesized OA-stabilized NPhs.

Material	K-edge	Attenuation	Bin	Solvent		Difference
OA-**Eu**	**48.5**	**0.627** ± 0.106	**bin 4**	**0.177** ± 0.011	30% EtOH	**0.450** ± 0.107
OA-**Gd**	**50.2**	**0.445** ± 0.021	**bin 4**	**0.177** ± 0.011	30% EtOH	**0.268** ± 0.024
OA-**Yb**	**61.3**	**0.374** ± 0.013	**bin 5**	**0.168** ± 0.007	30% EtOH	**0.206** ± 0.015
OA-**Lu**	**63.3**	**0.825** ± 0.087	**bin 5**	**0.168** ± 0.007	30% EtOH	**0.657** ± 0.087
OA-**Ta**	**67.4**	**1.188** ± 0.140	**bin 5**	**0.168** ± 0.007	30% EtOH	**1.020** ± 0.140
**Diatrizoate**	**33.2**	**0.475** ± 0.018	**bin 2**	**0.241** ± 0.007	100% water	**0.234** ± 0.019
**Material**	**K-edge**	**Attenuation**	**Bin**	**Solvent**		**Difference**
OA-**Gd/Eu** (50/50)	**48.5/50.2**	**0.404** ± 0.022	**bin 4**	**0.177** ± 0.011	30% EtOH	**0.227** ± 0.025
OA-**Yb/Lu** (50/50)	**61.3/63.3**	**0.309** ± 0.030	**bin 5**	**0.168** ± 0.007	30% EtOH	**0.141** ± 0.031
OA-**Yb/Eu** (98/2)	**61.3/48.5**	**0.341** ± 0.022	**bin 5**	**0.168** ± 0.007	30% EtOH	**0.173** ± 0.023
OA-**Yb/Er** (98/2)	**61.3/57.5**	**0.349** ± 0.012	**bin 5**	**0.168** ± 0.007	30% EtOH	**0.181** ± 0.014

Table S2. Attenuation coefficient values of different synthesized PAA-stabilized NPhs.

Material	K-edge	Attenuation	Bin	Solvent		Difference
PAA-**Eu**	**48.5**	0.270 ± 0.014	**bin 4**	**0.184** ± 0.015	100% water	**0.086** ± 0.021
PAA-**Gd**	**50.2**	0.212 ± 0.014	**bin 4**	**0.184** ± 0.015	100% water	**0.028** ± 0.021
PAA-**Yb**	**61.3**	0.200 ± 0.017	**bin 5**	**0.173** ± 0.010	100% water	**0.027** ± 0.020
PAA-**Lu**	**63.3**	0.209 ± 0.010	**bin 5**	**0.173** ± 0.010	100% water	**0.036** ± 0.014
PAA-**Ta**	**67.4**	0.202 ± 0.009	**bin 5**	**0.173** ± 0.010	100% water	**0.029** ± 0.013
**Diatrizoate**	**33.2**	**0.475** ± 0.018	**bin 2**	**0.241** ± 0.007	100% water	**0.234** ± 0.019
Material	K-edge	Attenuation	Bin	Solvent		Difference
PAA-**Gd/Eu** (50/50)	**48.5/50.2**	**0.227** ± 0.014	**bin 4**	**0.184** ± 0.015	100% water	**0.043** ± 0.021
PAA-**Yb/Lu** (50/50)	**61.3/63.3**	**0.182** ± 0.009	**bin 5**	**0.173** ± 0.010	100% water	**0.009** ± 0.013
PAA-**Yb/Eu** (98/2)	**61.3/48.5**	**0.178** ± 0.008	**bin 5**	**0.173** ± 0.010	100% water	**0.005** ± 0.013
PAA-**Yb/Er** (98/2)	**61.3/57.5**	**0.206** ± 0.011	**bin 5**	**0.173** ± 0.010	100% water	**0.033** ± 0.015
PAA-**Gd/Lu** (50/50)	**63.3/50.2**	**0.225** ± 0.015	**bin 4**	**0.184** ± 0.015	100% water	**0.041** ± 0.021

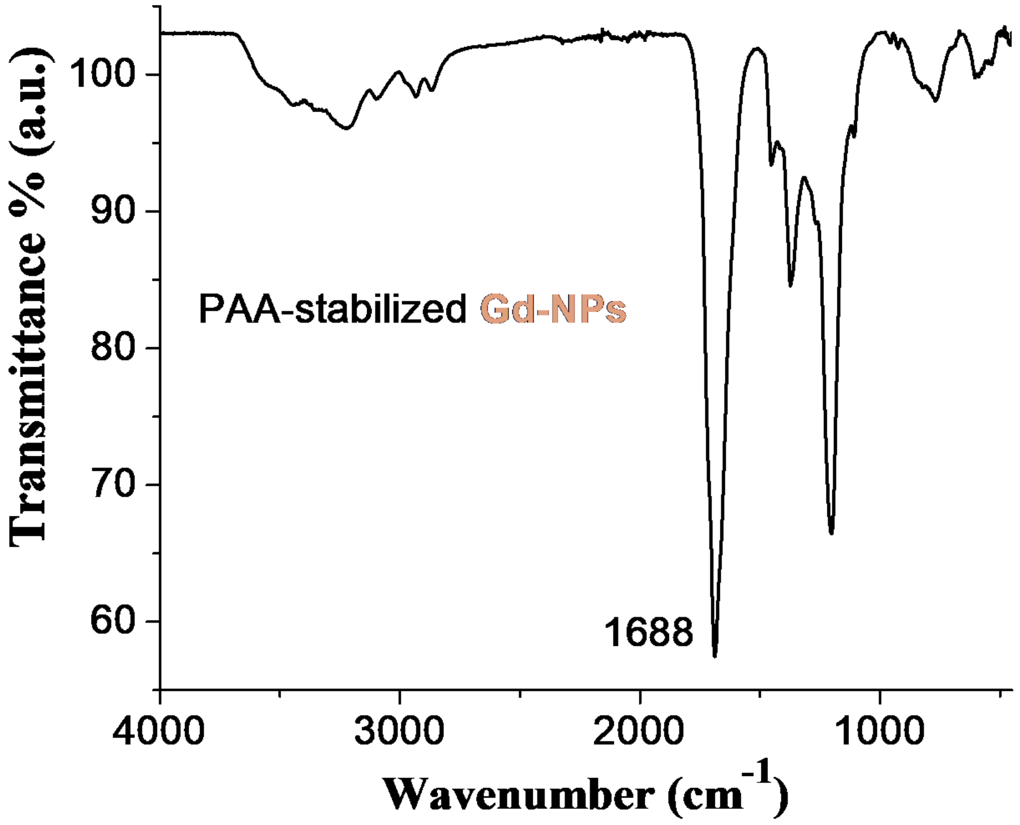

Figure S5. FTIR spectrum of PAA-stabilized Gd-NPhs.

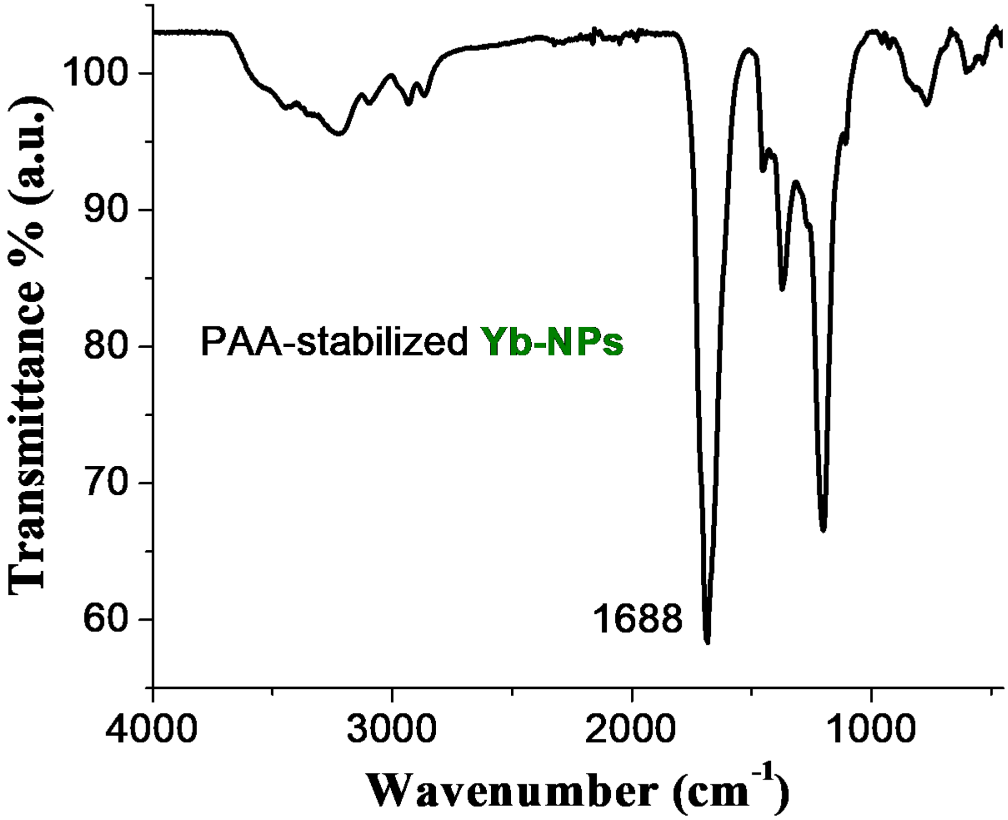

Figure S6. FTIR spectrum of PAA-stabilized Yb-NPhs.

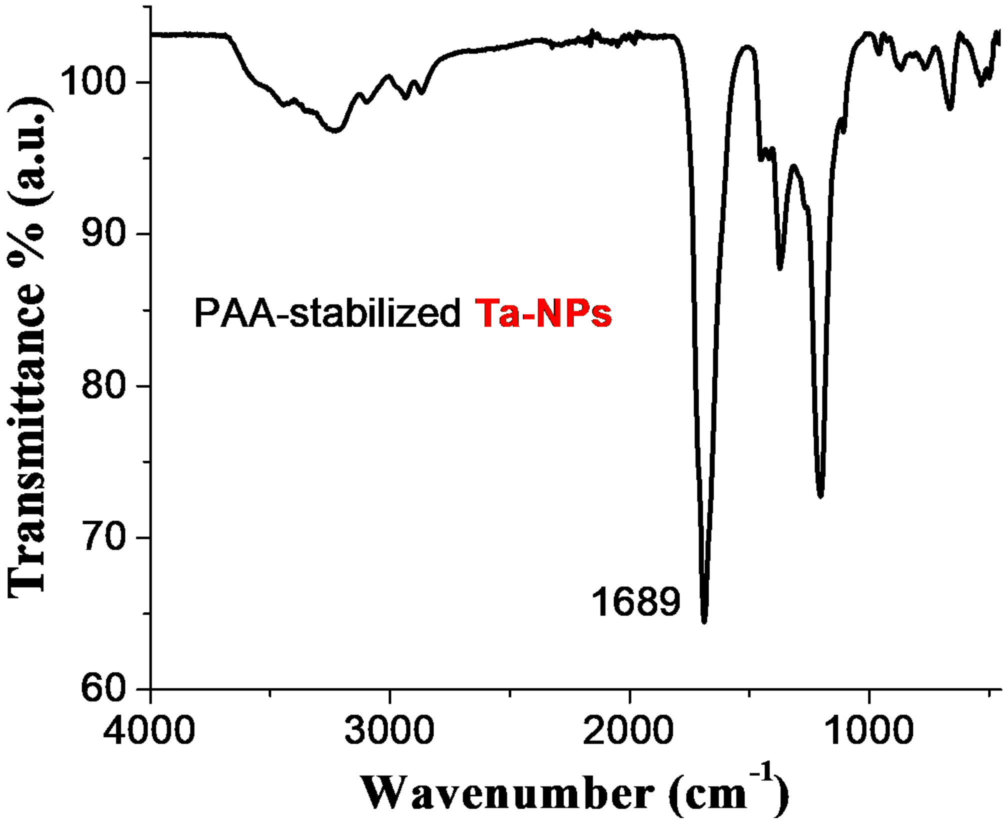

Figure S7. FTIR spectrum of PAA-stabilized Ta-NPhs.

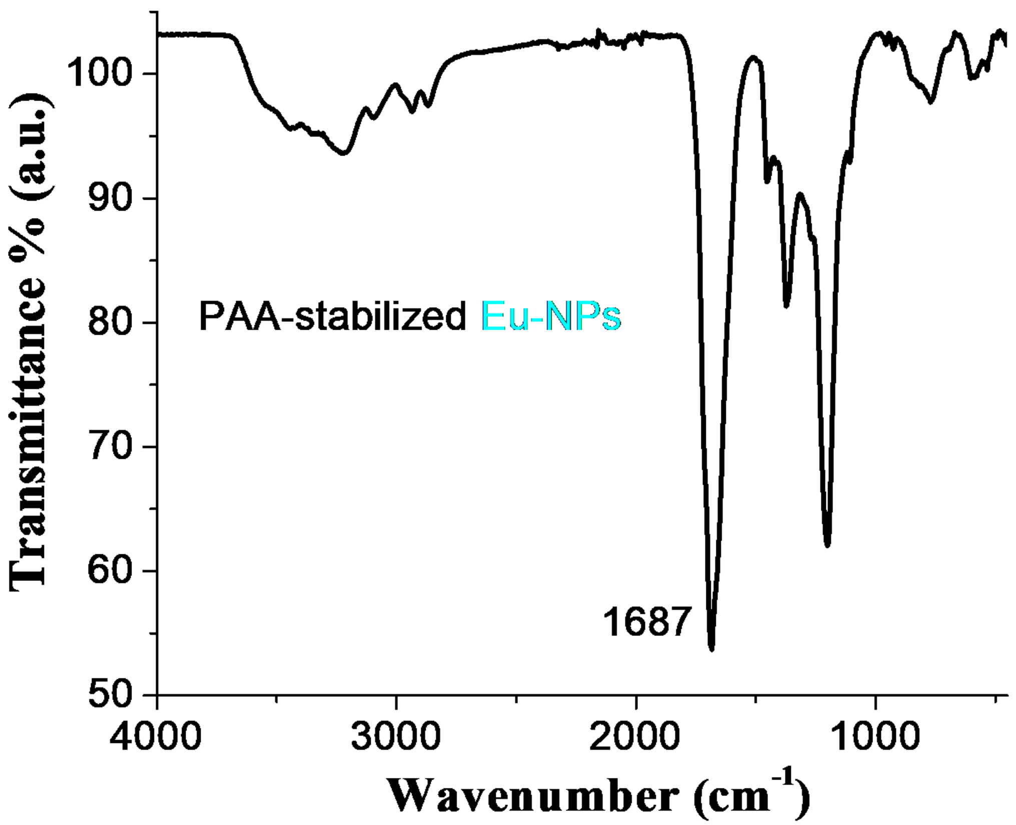

Figure S8. FTIR spectrum of PAA-stabilized Eu-NPhs.

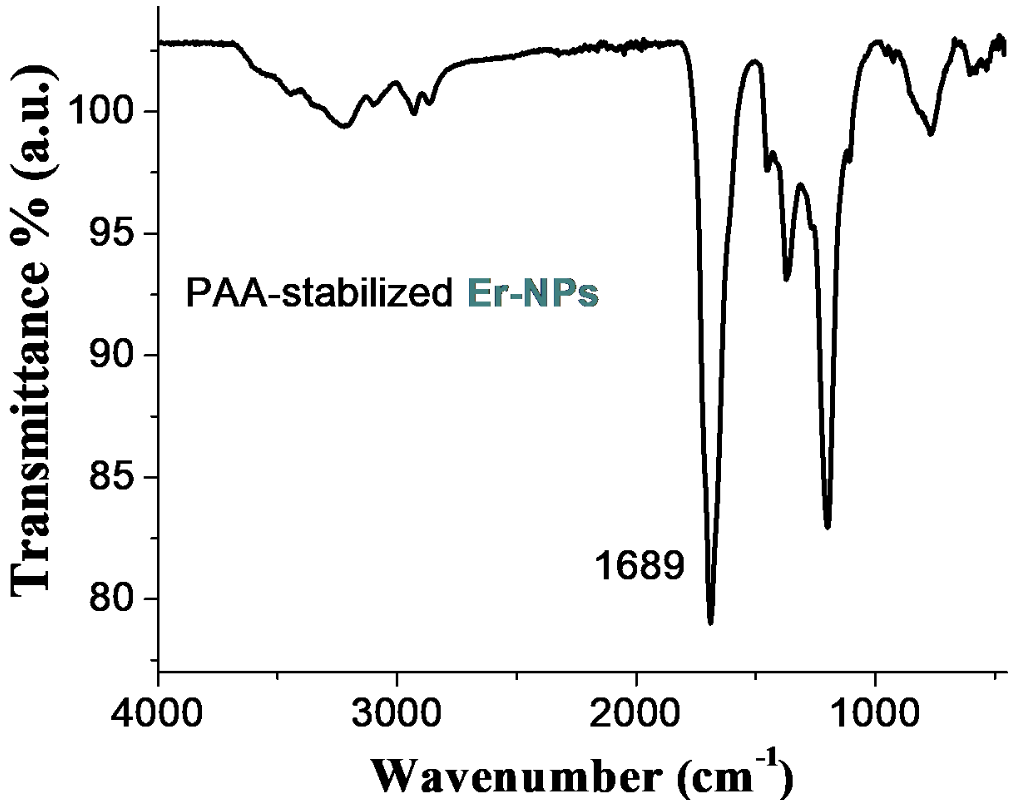

Figure S9. FTIR spectrum of PAA-stabilized Er-NPhs.

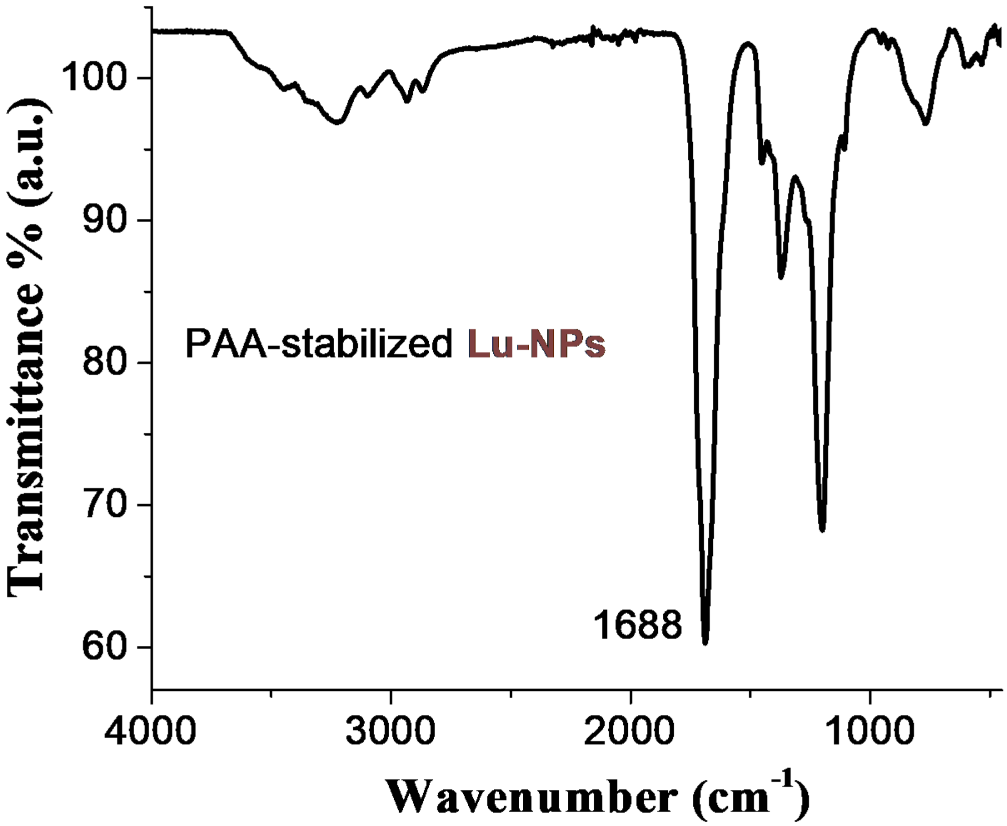

Figure S10. FTIR spectrum of PAA-stabilized Lu-NPhs.

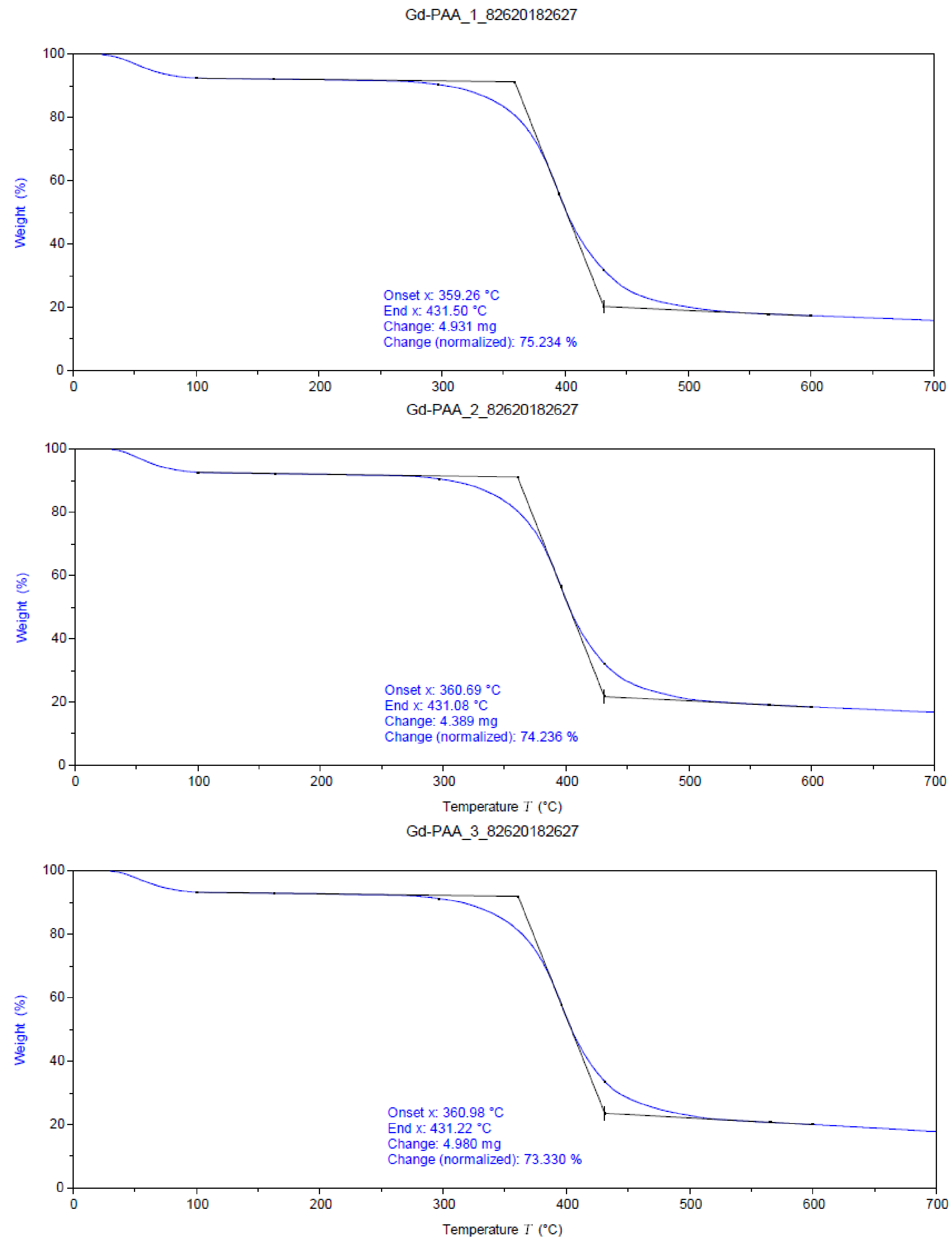

Figure S11. TGA spectra of $PAA_{1.8K}$-stabilized Gd-NPhs.

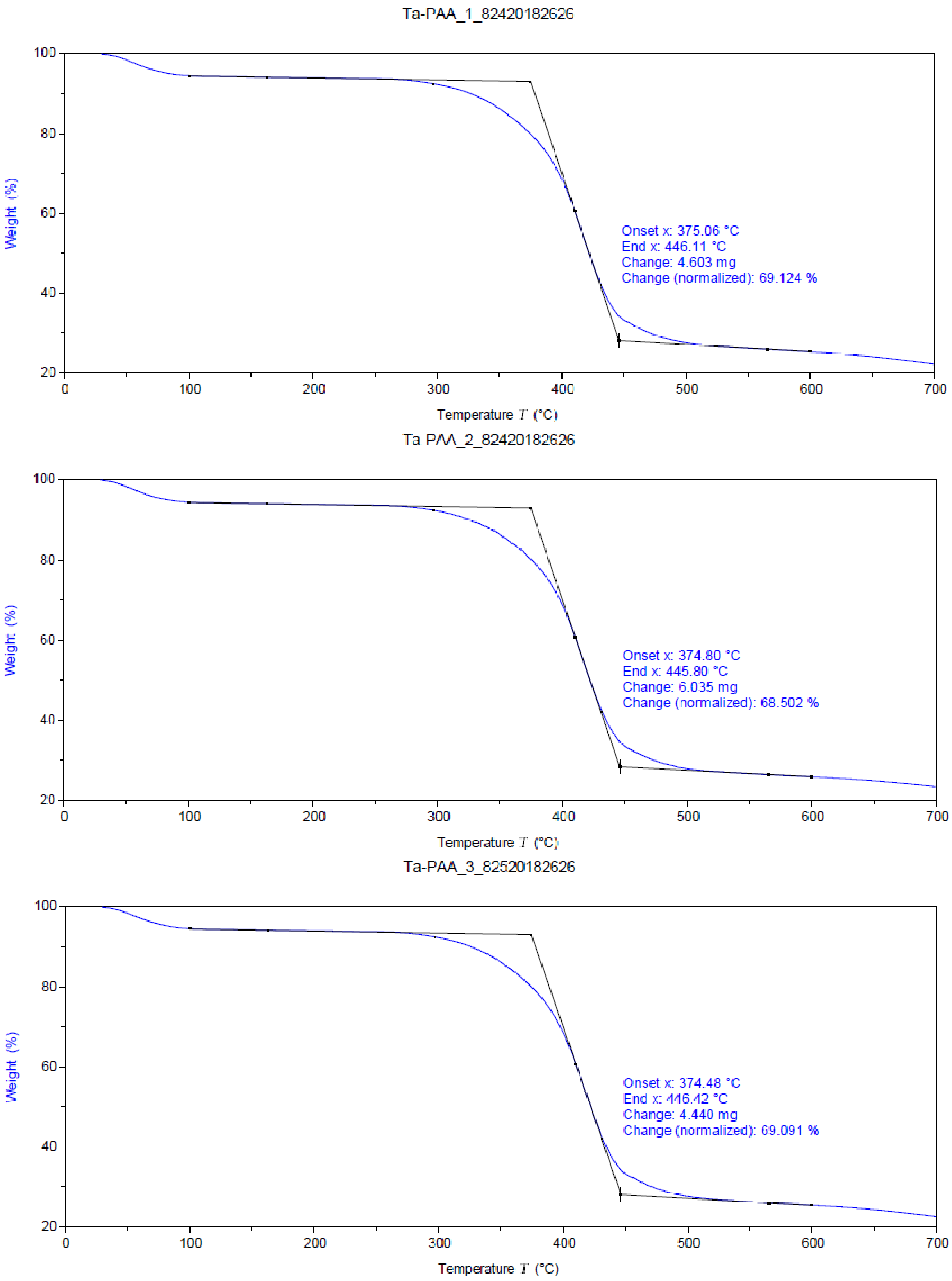

Figure S12. TGA spectra of $PAA_{1.8K}$-stabilized Ta-NPhs.

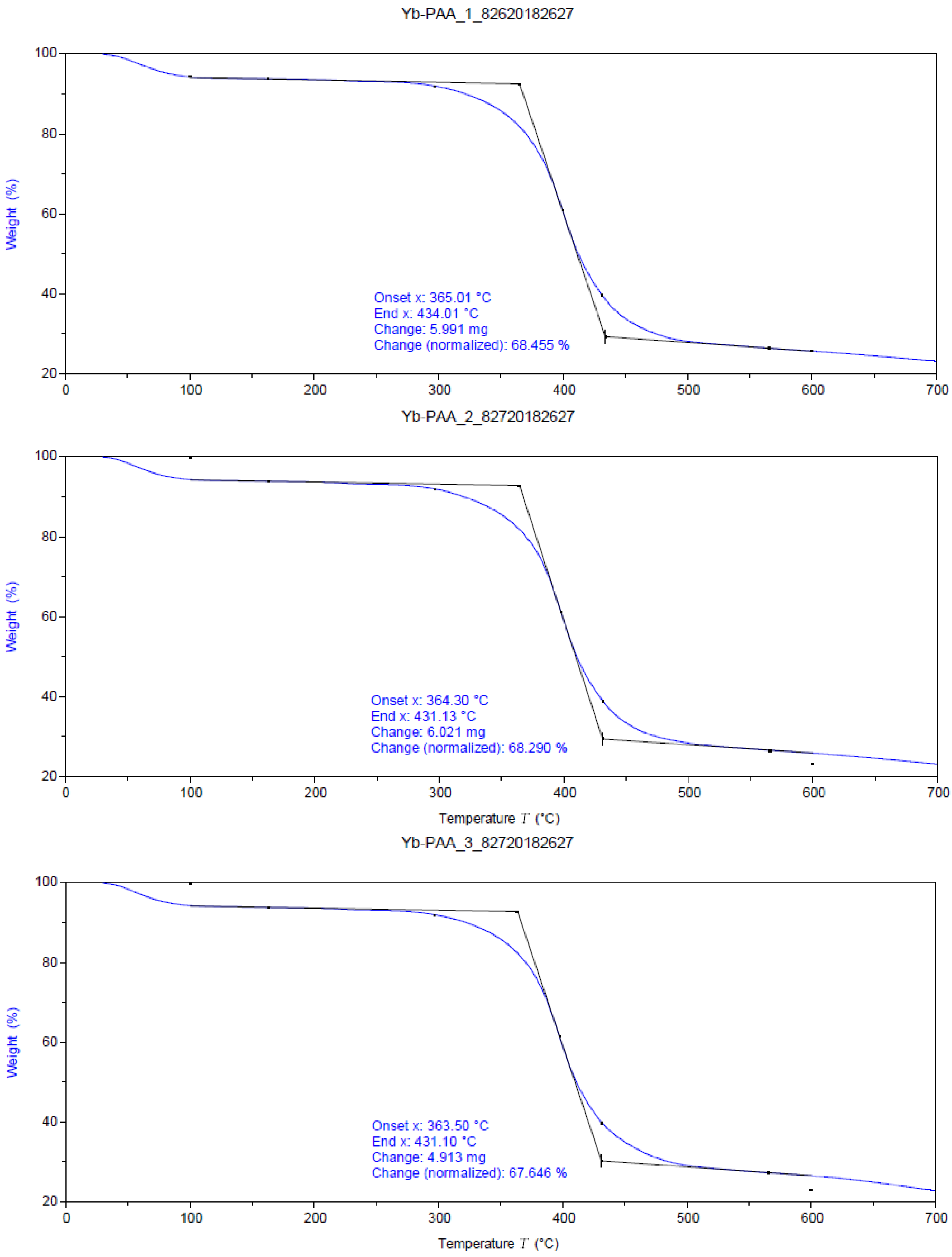

Figure S13. TGA spectra of $PAA_{1.8K}$-stabilized Yb-NPhs.

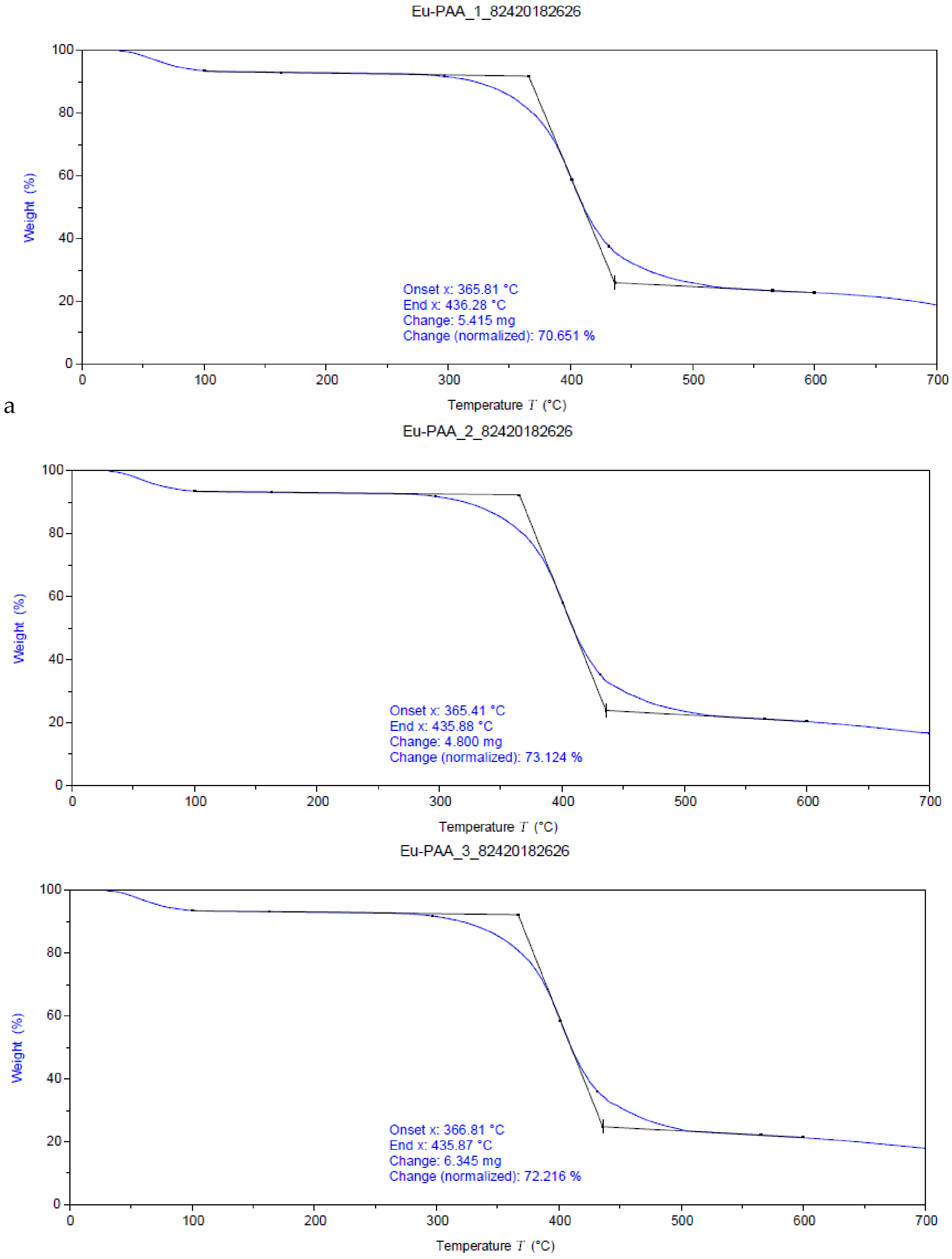

Figure S14. TGA spectra of $PAA_{1.8K}$-stabilized Eu-NPhs.

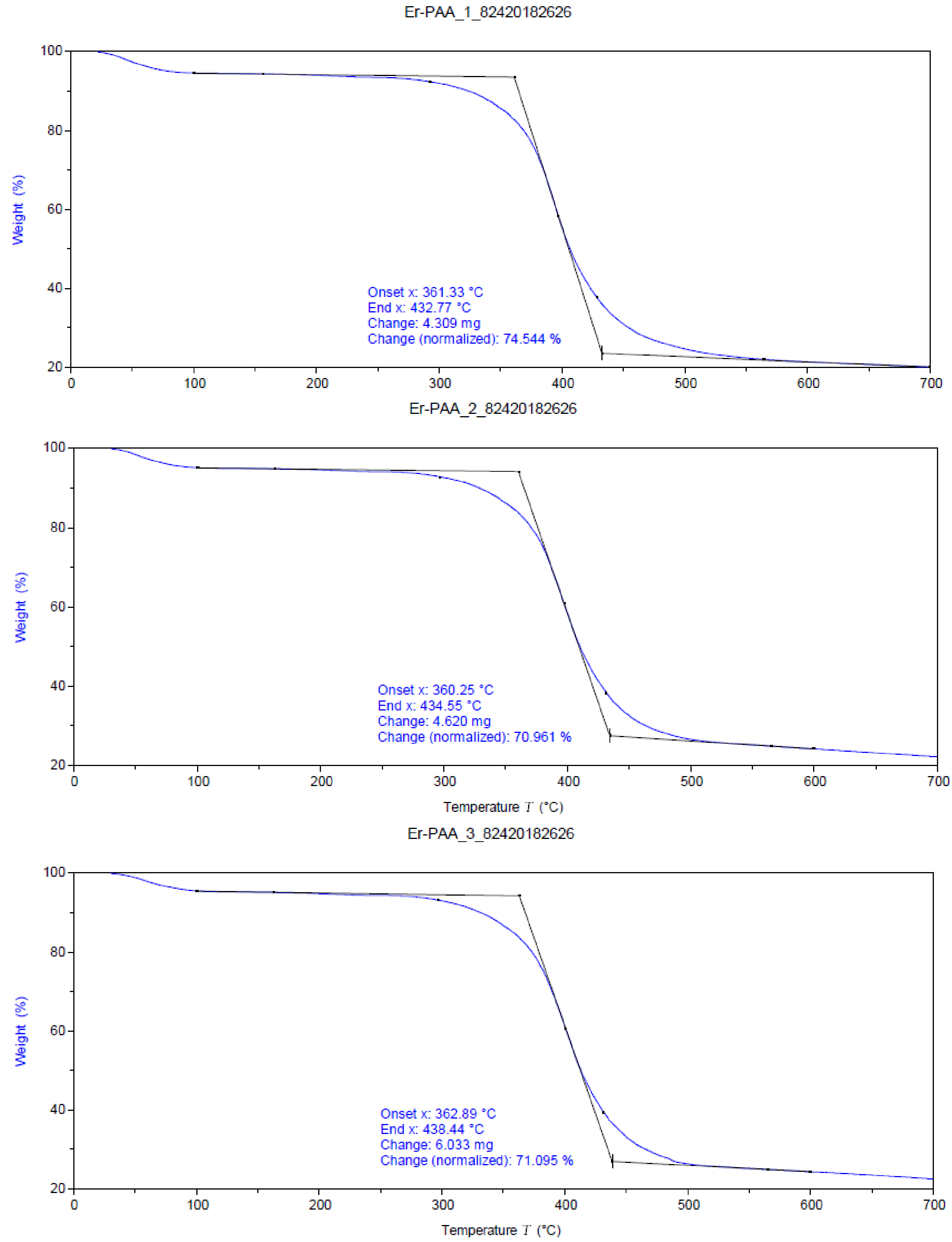

Figure S15. TGA spectra of $PAA_{1.8K}$-stabilized Er-NPhs.

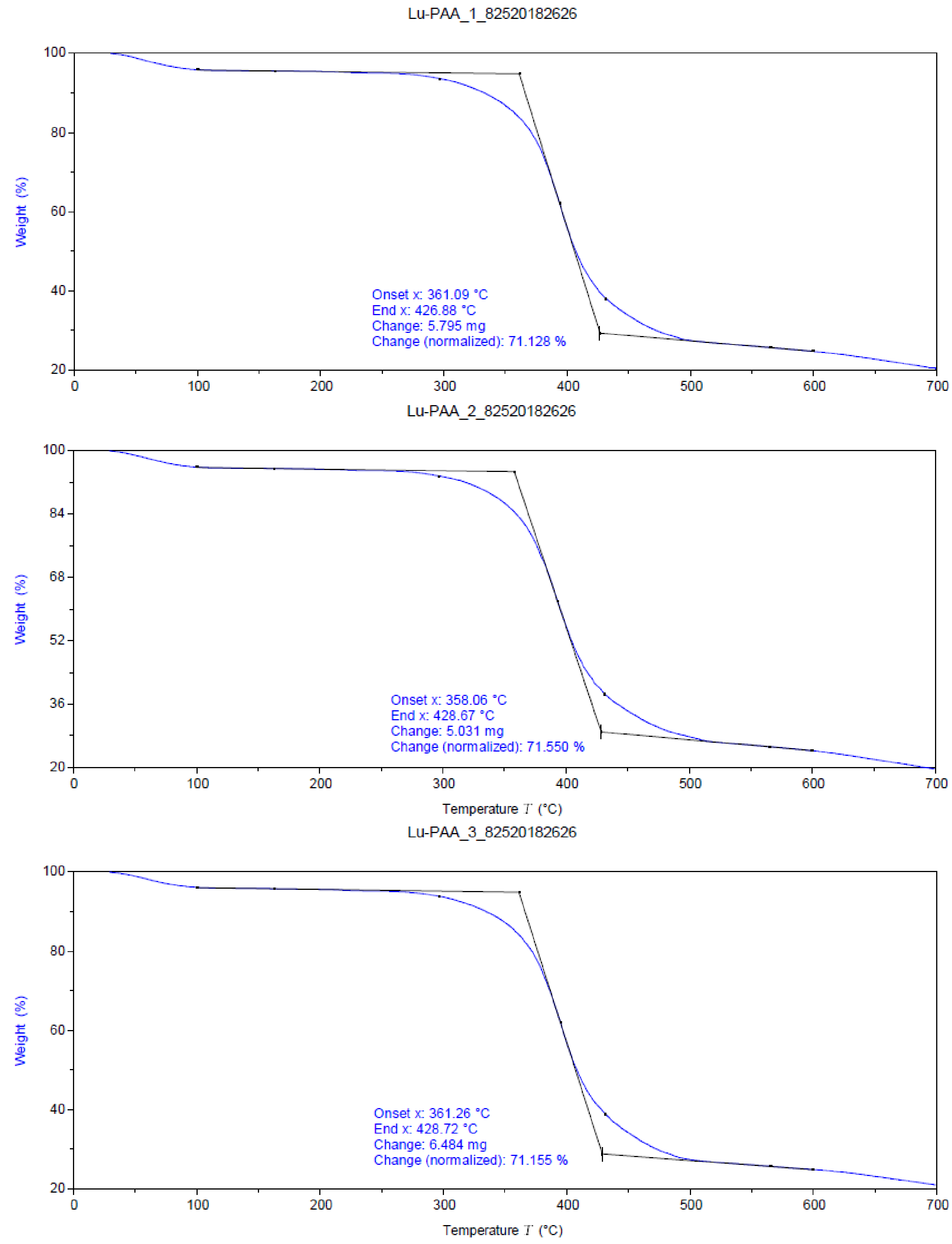

Figure S16. TGA spectra of $PAA_{1.8K}$-stabilized Lu-NPhs.

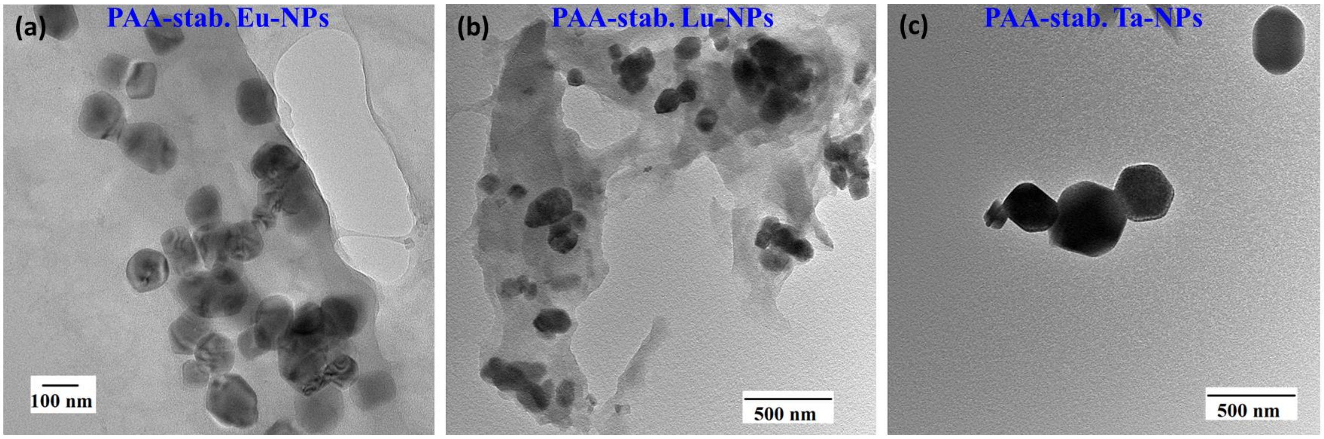

Figure S17. TEM images of PAA-stabilized (**a**) Eu-NPhs, (**b**) Lu-NPhs and (**c**) Ta-NPhs showing partial particle clustering after drying from water for the last two, with a tendency to form dimers or trimers. Therefore, DLS was used as a more accurate technique for size estimation in solution, showing a hydrodynamic diameter (z-average) of 216.0 ± 10.3 nm, 226.2 ± 15.1 nm, and 184.6 ± 6.8 nm for PAA-stabilized Eu-NPhs, Lu-NPhs, and Ta-NPhs respectively.

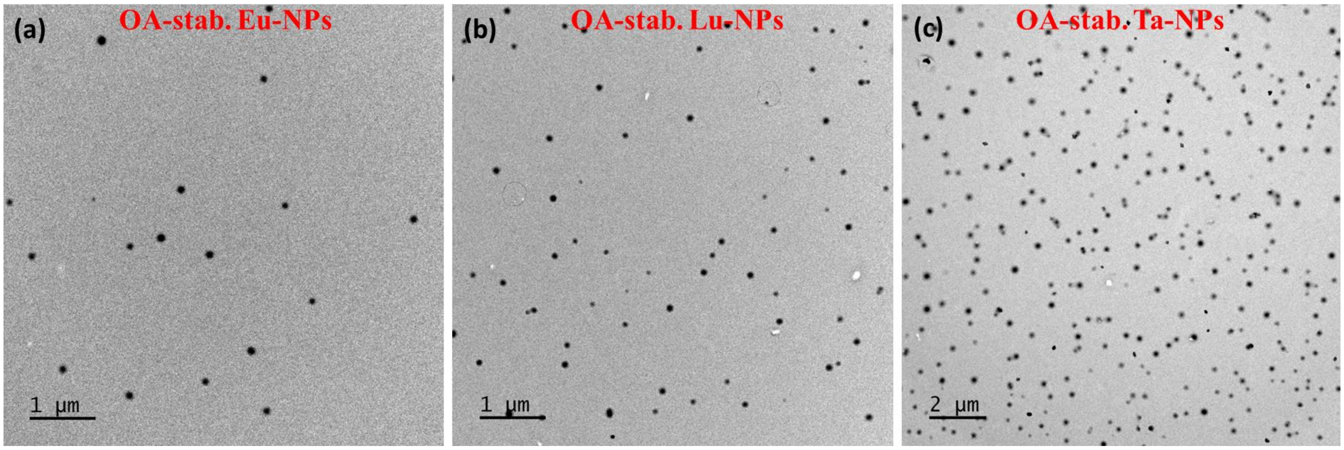

Figure S18. TEM characterization of OA-stabilized (**a**) Eu-NPhs, (**b**) Lu-NPhs, and (**c**) Ta-NPhs. Samples were prepared from ethanol.

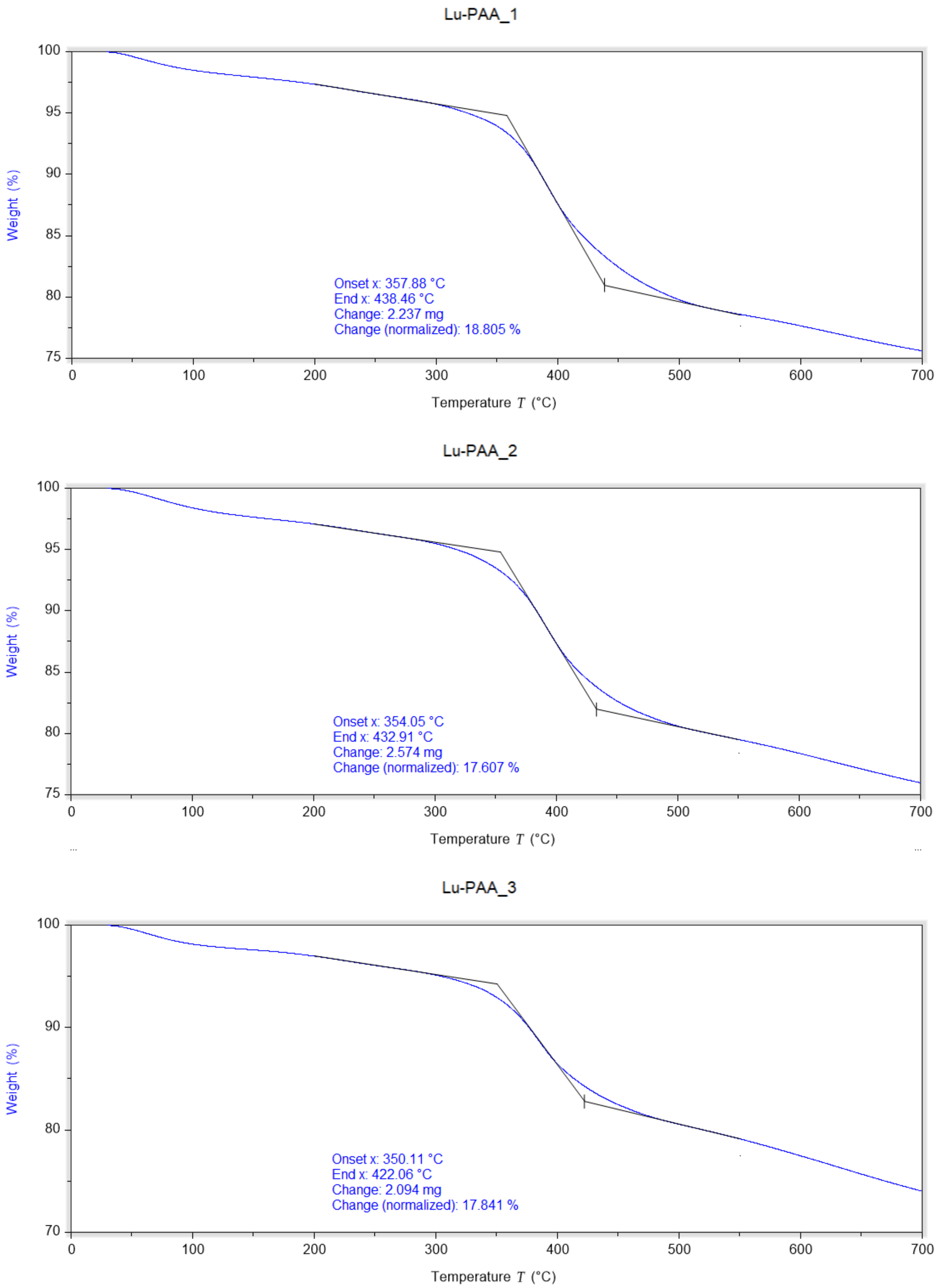

Figure S19. TGA spectra of newly synthesized PAA-stabilized Lu-NPs with a grafting density of 18.1% ± 0.6.

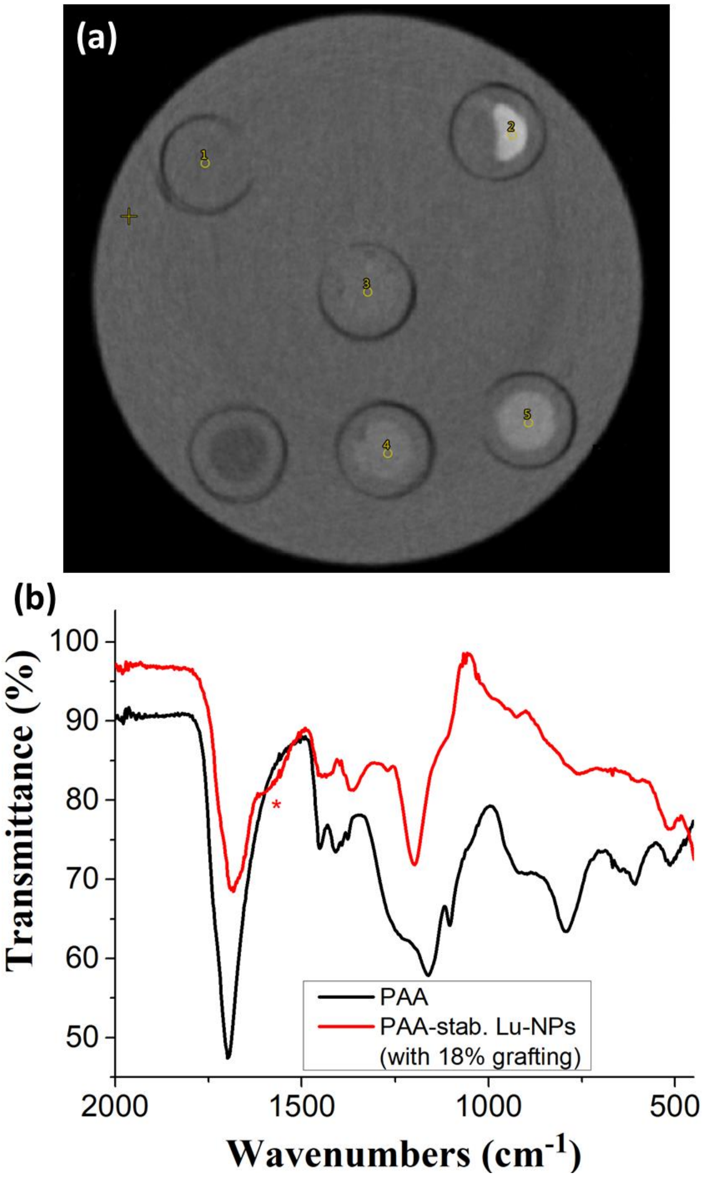

Figure S20. (**a**) Micro-CT image of PAA′-stabilized Lu-NPhs (labeled 2 in the image) at 90 mg/mL in water using the acrylic tubes shown in S2. The material with ~18% grafting density showed a high attenuation coefficient in the range of 0.303 ± 0.017 cm^{-1}, as well as moderate-to-good dispersibility. (**b**) FTIR spectrum of synthesized PAA′-stabilized Lu-NPhs. The characteristic C=O stretching peak for carboxylic acid, shown at 1695 cm^{-1} in the case of poly(acrylic acid) (black line, PAA), was slightly shifted to 1688 cm^{-1} for the case of new PAA-stabilized Lu-NPhs (red line), while the bound polymer (asterisk) onto the nanocrystal surface is shown at 1565 cm^{-1} (v_{as}: COO^-) and 1445 cm^{-1} (v_s: COO^-). Similar peaks have been also reported in the literature[48-50].
